# IoT Authentication in Federated Learning: Methods, Challenges, and Future Directions

**DOI:** 10.3390/s25247619

**Published:** 2025-12-16

**Authors:** Arwa Badhib, Suhair Alshehri, Asma Cherif

**Affiliations:** 1 Information Technology Department, Faculty of Computing and Information Technology, King Abdulaziz University, Jeddah 21589, Saudi Arabia; 2 Center of Excellence in Smart Environment Research, King Abdulaziz University, Jeddah 21589, Saudi Arabia

**Keywords:** IoT, authentication, federated learning, blockchain, behavior, biometric, cryptography

## Abstract

The Internet of Things (IoT) has established an exceptional ecosystem of interconnected devices where a vast multitude of heterogeneous devices can communicate, collect, and share data for enhanced decision-making processes. To effectively analyze this immense volume of data, researchers have deployed advanced machine learning algorithms and deep neural networks. However, these approaches typically rely on centralized data storage for training, which raises significant privacy concerns. Federated Learning (FL) addresses this issue by allowing devices to train local models on their own data and share only model updates. Despite this advantage, FL remains vulnerable to several security threats, including model poisoning, data manipulation, and Byzantine attacks. Therefore, robust and scalable authentication mechanisms are essential to ensure secure participation in FL environments. This study provides a comprehensive survey of authentication in FL. We examine the authentication process, discuss the associated key challenges, and analyze architectural considerations relevant to securing FL deployments. Existing authentication schemes are reviewed and evaluated in terms of their effectiveness, limitations, and practicality. To provide deeper insight, we classify these schemes along two dimensions as follows: their underlying enabling technologies, such as blockchain, cryptography, and AI-based methods, and the system contexts in which FL operates. Furthermore, we analyze the datasets and experimental environments used in current research, identify open research challenges, and highlight future research directions. To the best of our knowledge, this study presents the first structured and comprehensive analysis of authentication mechanisms in FL, offering a foundational reference for advancing secure and trustworthy federated learning systems.

## 1. Introduction

The Internet of Things (IoT) is a technology that connects billions of devices globally, which enables them to communicate and exchange data across diverse environments. According to the data analysis report of the International Data Corporation, IoT-connected devices will increase, reaching more than 41 billion devices and generating around 79.4 zettabytes of data [1,2]. This network integrates sensors, actuators, and smart devices into daily applications, which facilitates automation, improves efficiency, and generates large amounts of data for real-time analysis. IoT has an important impact on different sectors from healthcare and transportation to smart homes and industrial automation. For example, the profits from the smart home sector are growing rapidly, expected to reach around USD 96.7 billion (71.74%) between 2023 and 2028 [3]. Additionally, by 2028, the IoT market is estimated to reach a new peak of USD 231.6 billion, continuing its trend of consistent growth over the past years [3]. This massive amount of data from IoT needs to be processed effectively in order to make precise decisions. To achieve this, Machine Learning (ML) and Deep Learning (DL) techniques have been proposed as effective solutions [4,5]. However, using ML and DL algorithms requires all the data to be collected in a central server to perform accurate training. It also needs to combine large amounts of data to build up a high-quality model. This centralized approach raises significant concerns about privacy, security, and communication overhead, especially in large-scale networks such as IoT.

To overcome these challenges, Federated Learning (FL) is presented as a distributed solution. It allows training the ML model locally, without transferring sensitive data to a third party. It has been used in several domains to preserve privacy, such as smart cities [6], healthcare [7], vehicles [8], etc. Despite its privacy-preserving advantages, FL introduces new security challenges. One of the most critical concerns is ensuring the authenticity of participating devices and users. In dynamic and untrusted networks like IoT, traditional one-time authentication mechanisms, such as login-based credentials or static authentication, may be insufficient in ensuring ongoing trust during the session [9]. Devices can be compromised after initial access, and attackers can impersonate legitimate nodes or inject malicious model updates, which affects the integrity of the global model [10]. This requires robust authentication mechanisms to be developed. Second, traditional authentication schemes are not suitable for FL due to its decentralized, non-IID (Non-Independent and Identically Distributed) nature [11] as a result, new schemes need to be developed. Moreover, balancing robust authentication with user anonymity and untraceability is essential to maintain privacy, taking into account different attacks that threaten the authentication process, such as impersonation, Sybil, man-in-the-middle, session hijacking, etc.

Recently, several authentication schemes were developed in FL for different applications [8,12,13,14,15]. These schemes vary between static, continuous, multi-factor, and so on, utilizing several lightweight cryptography algorithms, blockchain, biometric, and behavioral. Most of these schemes were presented between 2023 and 2025, while only a few appeared in 2021 and 2022. The solutions vary between either utilizing federated learning to authenticate users/devices using behavioral or biometric data, without the need for large datasets to be collected in one device. This allows the data to be trained locally on each machine, while sending updates to improve the overall accuracy [16]. The other solution is to provide security for FL devices using several techniques, such as cryptography and blockchain.

Federated learning has gained significant attention, particularly concerning its security and privacy aspects. Several comprehensive surveys have been presented in the security field that focus on several aspects, such as the following:

- Security and privacy in FL [17,18,19,20,21]. These research surveys cover the security aspects of FL in terms of threats, vulnerabilities, and countermeasures, as well as privacy issues related to FL and suggestions for future research directions, emphasizing the balance between security and resource constraints.
- Blockchain FL [22,23,24,25,26,27,28,29,30]. These surveys concentrate on the integration of FL with blockchain, architecture, threats, challenges, how blockchain overcomes these challenges, limitations, and possible future directions for this integration.
- FL for Intrusion detection systems [31]. It focuses on applying FL to develop an IDS that can detect anomalies and malicious activities across distributed networks without centralizing sensitive data.
- Trustworthy FL [32,33]. These surveys examine the principles and frameworks necessary to establish trust in FL systems. They also cover different aspects such as security, robustness, and privacy, proposing comprehensive road maps for developing trustworthy FL. The discussion includes threat analyses and defense strategies to ensure the reliability of FL deployments.
- FL security for healthcare [34,35,36,37,38]. These surveys presented a detailed review of Internet of Medical Things (IoMT), including device types, security issues, attacks, and security mechanisms. They also highlighted how FL enhances the privacy and security of the healthcare sector and devices.
- FL for biometric recognition [16]. There is only one survey in this area that concentrates on how FL can be integrated into the biometric recognition field to enhance the accuracy and privacy of the systems. It also discusses the future directions, challenges, and opportunities in this area.

**Figure 1:**
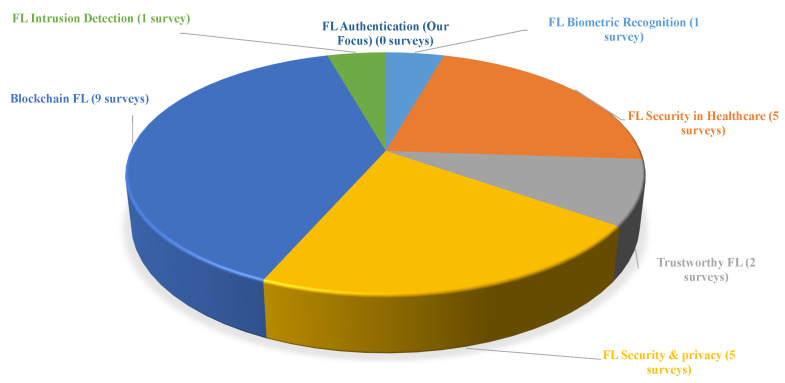
Distribution of FL survey research across domains.

All the mentioned surveys cover the security and privacy aspects in general and did not concentrate on authentication in FL or compare their schemes. Figure 1 shows the distribution of FL surveys across various domains with the number of research in each area. The blockchain integration into FL has the highest number, with eight research works. This visualized representation also shows that there is a notable gap in comprehensive surveys focusing specifically on authentication mechanisms within FL systems. To the best of our knowledge, this is the first survey in this direction. As we discuss how authentication is implemented in FL, we review and discuss in detail the current authentication mechanisms and compare them according to defined criteria. These schemes are analyzed and classified based on the following two dimensions:

- The underlying technology these schemes use. This category discusses three main technologies, outlined as follows: Blockchain-based, cryptography-based, and Artificial Intelligence (AI)-based.
- The system context in which FL is deployed. This category is also divided into three main parts as follows: cross-silo, cross-device, and hybrid approach.

Additionally, we discussed all the challenges, architecture, and future directions in this area.

### 1.1. Research Objectives

Given the gap in the literature on federated learning authentication, we define our objectives for this comparative analysis research as follows:

- Evaluate the current mechanisms in FL authentication and examine their effectiveness and limitations.
- Identify challenges and vulnerabilities within authentication schemes that may compromise security or efficiency.
- Propose a set of criteria for designing robust authentication methods tailored to the unique requirements of FL systems.
- Analyze the influence of the design of the system context in FL on the development of the authentication schemes.
- Suggest potential future directions for further investigation to enhance authentication in FL, considering emerging technologies.

According to these objectives, we formulate key research questions to guide our analysis and literature review, ensuring a structured and comprehensive investigation.

### 1.2. Research Questions

In order to address the challenges and gaps in authentication for federated learning, we formulate the following research questions. The following questions direct our analysis, enabling us to evaluate existing authentication mechanisms, identify limitations, and propose future research directions in this field:

RQ1: How is authentication integrated into federated learning in terms of the process, challenges, and architecture?

RQ2: What are the existing underlying technologies used to authenticate users/devices in federated learning?

RQ3: How does the system context in federated learning affect the development of the authentication scheme?

RQ4: What open research challenges remain in FL authentication, and what are the promising future directions to enhance authentication mechanisms?

Based on these questions and objectives, we come up with our research contributions.

### 1.3. Research Contributions

To answer these questions and address the gaps in the literature on authentication in FL, we conduct a comparative analysis of current authentication mechanisms in FL, evaluate their effectiveness and limitations, and propose future directions to enhance security in decentralized learning environments. We believe that this research will act as a guide for researchers who are interested in developing this area. Our contributions can be summarized as follows:

- We provide a comprehensive overview of the fundamental aspects of authentication in FL, defining the process, different architectural frameworks, and the challenges related to this field.
- We propose a taxonomy to classify the existing literature based on the technologies used, distinguishing between blockchain, cryptographic algorithms, and behavioral/biometric machine learning techniques. Based on this classification, we review, compare, and analyze the limitations of each authentication scheme.
- We analyze and classify the authentication schemes in federated learning from another perspective based on the system context in which they operate, particularly focusing on cross-device, cross-silo, and hybrid settings. This highlights how deployment characteristics influence the authentication requirements and design.
- We also provide an overview of the datasets used in FL authentication schemes, discussing their relation to authentication type and their ability to reflect realistic FL challenges. This will offer a realistic and practical guide for researchers in selecting appropriate datasets when designing and evaluating their authentication schemes.
- Finally, we highlight the open issues and future directions related to authentication in FL, and we provide some recommendations that can guide researchers to improve the field.

### 1.4. Survey’s Structure

The aim of this study is to propose a comprehensive research that presents the challenges, open issues, and future directions for authentication in FL, by answering the research questions. The research is organized as follows: Section 2 provides a background of authentication and federated learning, discussing their types and data partitioning. Section 3 provides an overview of authentication in FL, clarifying the authentication process, defining the challenges and threats related to the field, and examining various architectural models. Section 4 summarizes in detail the recent blockchain-based authentication schemes in FL, classifies them based on their blockchain type, and compares these schemes based on defined security metrics. Section 5 summarizes in detail the recent cryptography-based authentication schemes in FL, classifies them based on whether they are using Public Key Infrastructure (PKI) or certificateless, then compares these schemes based on defined security metrics. Section 6 discusses in detail the FL authentication schemes that are based on biometric and behavior data and compares them. Section 7 describes the influence of system context over the authentication schemes and their impact on the designing process. Section 8, analyzes the findings from the comparative analysis, highlighting key insights, strengths, and limitations of existing authentication mechanisms. Section 9 discusses the datasets used in the current literature. Finally, Section 10 provides open issues and possible future directions to improve this area and provides the conclusion.

## 2. Background

In this section, we clarify several primary aspects related to this research. We start by introducing the concept of authentication and its types, followed by an overview of federated learning and data partitioning in FL. Finally, we introduce post-quantum cryptography, its main categories, and its algorithms.

### 2.1. Authentication

Authentication is one of the most important security aspects that ensures that only legitimate users/devices join the system. The advent of new technologies requires the continuous improvement and development of the authentication process. For example, the advent of IoT required that authentication mechanisms be lightweight and suitable for these resource-constrained devices. Traditional password-based systems are increasingly inadequate, particularly for new technologies, and require robust security solutions that can mitigate threats related to the domain. Additionally, the development of a distributed machine learning approach (federated learning) required different authentication mechanisms to be improved rather than traditional ones. Considering the main challenges related to this field and according to this research, authentication can be classified into static authentication and continuous authentication based on the timing and frequency of identity verification. Static authentication is a one-time verification process that occurs at the login or session, where the user proves their identity using methods such as passwords, biometrics, or cryptographic certificates. There is a special type that falls under this category, which is a multi-factor authentication. This type integrates two or more authentication factors (e.g., password and OTP, fingerprint, and smart card), which is carried out at the beginning of each session [39]. On the other hand, continuous authentication goes beyond initial verification, as it continuously monitors users during the session. This type usually uses techniques, such as behavioral biometrics (keystroke dynamics and mouse movement), session monitoring, and risk-based adaptive authentication, to enable real-time identity validation and anomaly detection [9].

### 2.2. Federated Learning

Federated Learning is considered a transformation technology from a traditional centralized machine learning approach into a distributed approach. This allows multiple clients or devices to collaborate in the training process, without revealing any private or confidential data. It was first introduced by Google in 2016 [11] as a novel approach to train machine learning models across decentralized devices. Furthermore, it was initially applied in Google’s Gboard keyboard for personalized text prediction without compromising user data. The first method, FedAvg, was proposed by McMahan et al. [11] for the aggregation process. FedAvg aggregates all model parameters from the clients by computing a weighted average of their locally trained models. Recently, several research concentrated on FL and its integration with several domains, especially due to private regulations that restrict the transfer of sensitive data between different sites. To better understand how FL preserves privacy, we sum up the training steps as follows:

1. The server will choose the appropriate machine/deep learning approach for training and broadcast it among the clients, together with the initial parameter. This initial parameters can be a new trained model, randomized, or a pre-trained model.
2. Each client will receive this global parameter and start training their own local model using their own datasets. During training, the client calculates model updates (e.g., gradients or weight adjustments) based on its unique data distribution.
3. The clients start training their own local model using their own datasets. During training, the client calculates the model’s updates (e.g., gradients or weight adjustments) based on its unique data distribution.
4. Once the client completes the training, it sends its updated parameters to the server.
5. The server aggregates the received updates from all clients to refine the global model.

**Figure 2:**
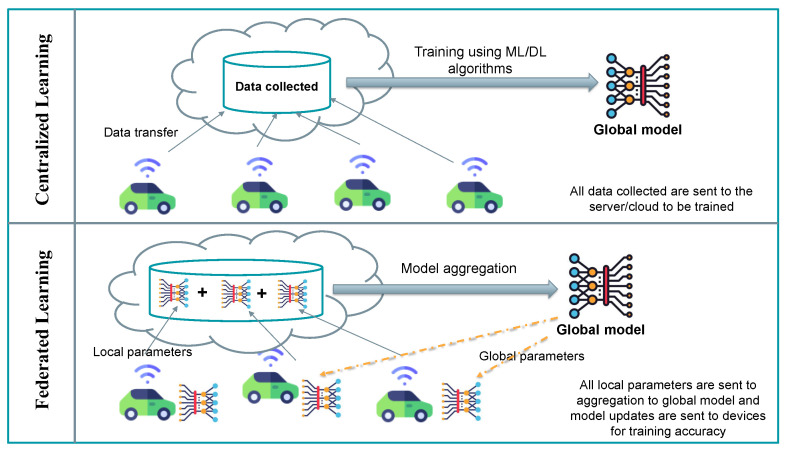
Centralized vs. federated learning.

These steps will be repeated until the model converges or reaches a specific predetermined threshold. Figure 2 illustrates the differences between the centralized approach and FL approach, as well as clarifies the FL training process. Recently, multiple frameworks and tools have been developed to implement FL practically. Some of these well-known tools and libraries are PyTorch 2.4.0 (https://pytorch.org/ (accessed on 10 June 2025)), TensorFlow Federated 0.88.0 (https://www.tensorflow.org/federated (accessed on 10 June 2025)), PySyft 0.9.5 (https://github.com/OpenMined/PySyft (accessed on 20 July 2025)), etc.

#### Data Partitioning in FL

**Figure 3:**
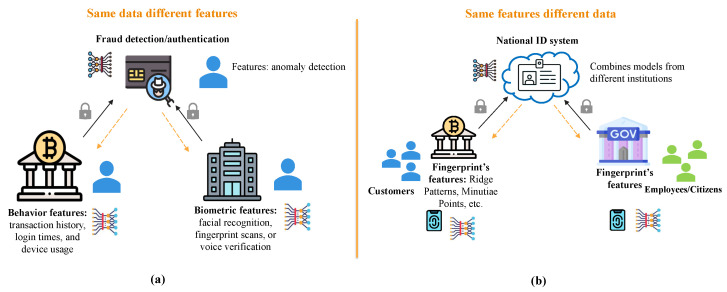
(**a**) Vertical FL. (**b**) Horizontal FL.

**Figure 4:**
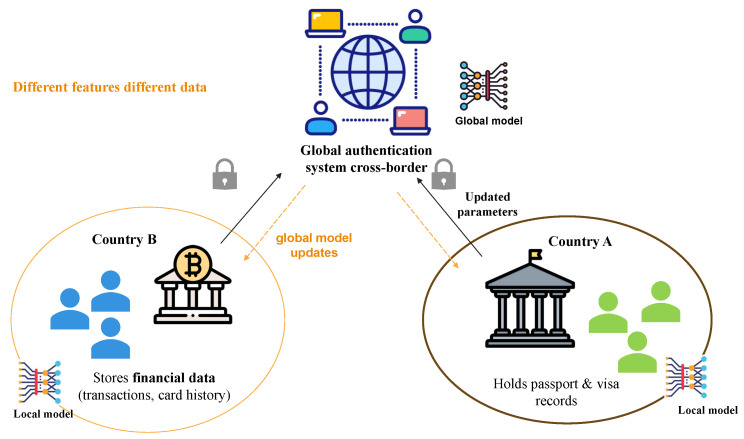
Transfer FL.

Based on the distribution of the data in terms of data space and feature space, FL can be divided into three main categories as follows: vertical, horizontal, and transfer learning. Each category is explained as follows:

1. Vertical FL: this type of data distribution is used when different clients have datasets that share the same users (samples) but different feature spaces [32]. The structure is illustrated in Figure 3a. For example, if both a bank and a technology company have overlapping users but collect different authentication data, the bank will track financial behaviors (i.e., transaction history, login times, and device usage), while the company collects biometric authentication data (i.e., facial recognition, fingerprint scans, or voice verification). Vertical FL allows both sectors to participate in training the global fraud detection/authentication model. This type of training is useful for combining complementary features from different domains, plus it improves predictive accuracy in behavior analysis and fraud detection.
2. Horizontal FL: This type of data distribution is used when multiple users have different data spaces and share the same feature space [14]. The structure of horizontal FL is presented in Figure 3b. This model is common in biometric authentication, where the features are the same for different users. For example, a government database and a bank both use fingerprints for authentication with the same features, but they store data for different people.
3. Federated transfer learning: This type of data distribution is used when both users (data samples) and features are different. This case will be useful if two organizations have different authentication data types and only a limited set of shared users. This data distribution is shown in Figure 4. For example, it can authenticate international cross-border users, if a bank in a certain country stores financial data, like transactions and card history. However, if an agency in another country holds passport and visa records, both authentication data can help in training a system that can detect international data without sharing private data.

Understanding these types of data distributions can help researchers to evaluate and build up their authentication model. Authentication applications depend on diverse data sources, especially if we are dealing with biometric/behavior approaches. The appropriate architecture will be selected according to the data partitions in the system.

## 3. Authentication in Federated Learning

Federated Learning is a technology that enables collaborative model training across decentralized devices while preserving data privacy. Traditional mechanisms may not be suitable for FL, due to its decentralized nature, heterogeneous devices, and privacy-preserving constraints. Accordingly, many research works focus on developing new authentication frameworks specific to FL. Authentication in FL has recently gained attention, with a significant increase in research publications in the last few years (2023–2025). While some initial studies emerged in 2021 and 2022, the rapid growth of FL applications has driven the need for enhanced authentication mechanisms, making this an active and evolving research domain. The proposed solutions cover several IoT domains, such as Internet of Vehicles (IoV) [8,13], Unmanned Aerial Vehicle (UAV) [14,40], Vehicular Ad-Hoc Network (VANET) [41,42], and IoMT [12,39].

On the other hand, from our observation, research in this area is moving towards two main directions. The first direction is using the FL approach to improve the accuracy of the authentication scheme. This is usually related to biometric/behavior-based authentication, as it helps in preserving privacy while enriching the training process. Several research works provide this direction such as [9,10,12,15,40,43]. The second direction is to ensure that all devices/users in the FL network are authentic and verified. This approach mainly utilizes blockchain, public-key cryptography, certificateless cryptography, zero-knowledge proof, and digital signature techniques. Several research works utilize this approach, such as [13,14,39,42,44,45,46,47,48].

**Figure 5:**
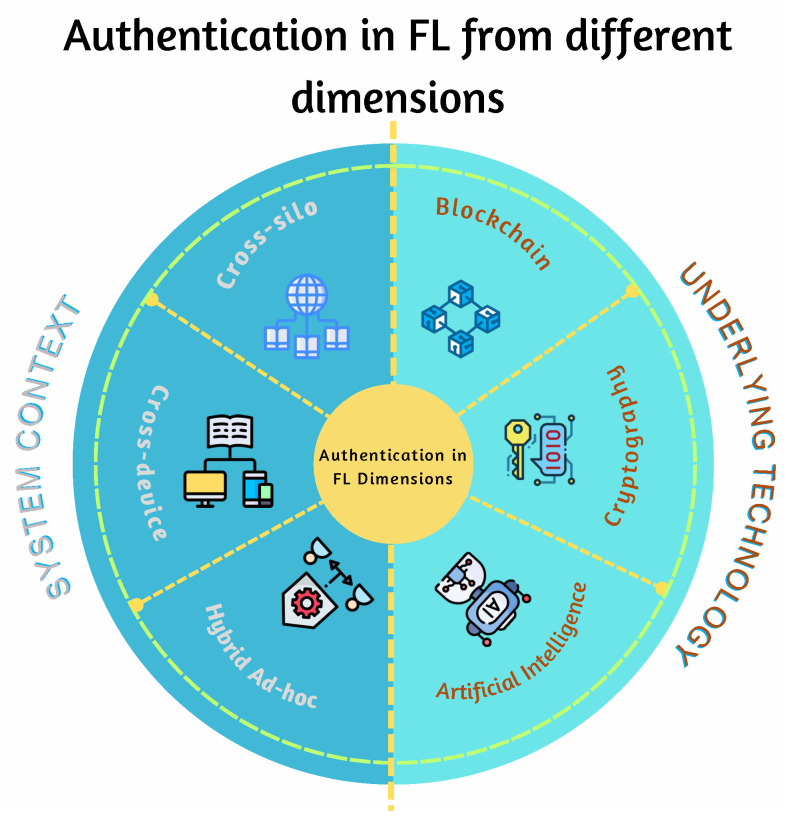
Authentication in FL dimensions.

Another key observation is that system context affects how authentication is implemented. We take into account that authentication mechanisms are also affected by the nature of the participating clients, system architecture, data distribution, and application domain. We found that there are three FL settings, namely, cross-silo [49,50,51], cross-device [52,53,54,55], and hybrid (ad hoc silo) [41,56,57]. Figure 5 shows the two dimensions in which the field authentication in FL can be categorized. These two perspectives provide a structured method to analyze schemes in this domain and identify the gaps, patterns, and trends in the literature. Based on this foundation, we categorized and analyzed the authentication mechanisms in FL.

Additionally, a new direction can be further investigated, which concentrates on the challenges related to clients’ selection during authentication. Only one research is presented by Wazzeh et al. [10], as it proposed continuous authentication based on federated and split learning. It concentrated on addressing the challenges related to client’s selection during split FL, taking into account the resource capacities of devices participating in the training process. It also optimizes client selection by using Genetic Algorithm (GA) to prioritize clients based on several factors, including their resource capacities, data sample sizes, processing capabilities, and unique labels.

### 3.1. Authentication Process

**Figure 6:**
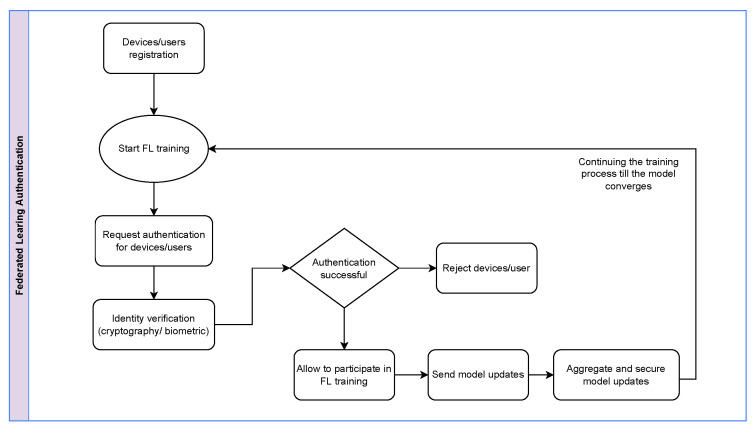
Authentication process in FL.

The general structure for the process of authentication in FL follows several steps that are shown in Figure 6. The authentication process begins with a registration in the system to ensure that only authentic devices join the network. After that, devices/users are authenticated, either before or during the FL training process. The authentication process begins with identity verification, using device-based, cryptography, biometric/behavior, or any other credentials that the system defines. Therefore, authentication can be managed according to the system’s architecture, using a centralized, decentralized, or blockchain server. If the authentication fails, then the device/user is rejected from the training process. This ensures that only verified users are updating the model. Otherwise, devices/users can grant access to send updated local parameters to train the global model. Note that this process will prevent multiple security threats, such as Sybil attacks, malicious client injections, etc.

### 3.2. Architecture

**Figure 7:**
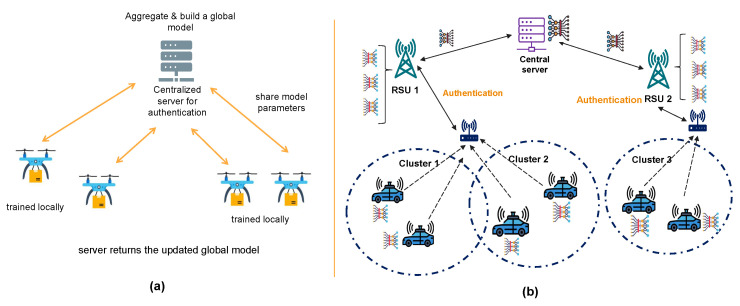
(**a**) Centralized FL. (**b**) Hierarchal FL.

**Figure 8:**
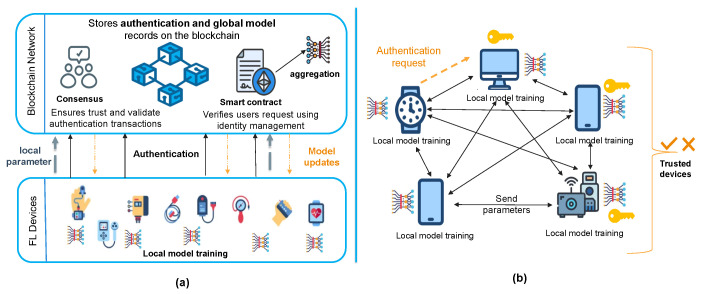
(**a**) Blockchain FL. (**b**) Peer-to-peer FL.

The architecture of authentication in FL can be categorized into several types, according to the authentication and aggregation operations. We classified them as follows:

1. Centralized FL: It is based on the general architecture of FL, where there is a centralized server that manages authentication and verifies all FL participants. It also performs the aggregation process and issues credentials, certificates, or authentication tokens to registered users/devices. It is a simple well-known approach; however, it suffers from a single point of failure and scalability issues. Figure 7a shows the details of this architecture [32]. Several studies employed this architecture, such as [9,12,15,40].
2. Hierarchical (partially distributed) FL: It is based on a multiple-layer structure, where multiple nodes are used to authenticate and aggregate. These nodes are organized as several clusters or regions, and each cluster has a cluster head that is responsible for verifying users and combining parameters. After that, authentic aggregated parameters can be sent to the server to update the global model [42]. This approach provides more scalability and flexibility while reducing communication overhead on the server. However, communication between the nodes and their cluster head needs to be secure. Also, managing the movement from one cluster to another needs to be considered. Figure 7b illustrates this type of architecture using an IoV scenario, where the authentication process is usually performed by the Road-Side Units (RSUs). As a future direction, research can investigate more distributed authentication methods towards the edges. Multiple authentication solutions utilize this architecture, such as [39,42].
3. Distributed FL using blockchain: It is based on a distributed architecture, using blockchain rather than depending on a single server. The authentication process is handled on a blockchain, ensuring tamper-proof, decentralized identity verification [58]. Blockchain can establish trust among clients using a consensus algorithm. Additionally, it can be used for incentives and rewards to encourage trusted clients and improve data model quality. Smart contracts automate authentication rules, enforce access policies, and verify participant identities without human intervention [28]. Several mechanisms employed this approach, such as [13,41,44,45]. Figure 8a presents the general architecture of this type of frameworks.
4. Peer-to-peer FL (fully distributed): It is based on direct communications between users. It does not depend on any centralized server. This approach has not been considered in authentication in FL, and to the best of our knowledge, there is limited elaboration in authentication using peer-to-peer FL. The only work found is presented in [49], which presents a continuous authentication mechanism in federated machine learning. It is based on collecting and analyzing behavioral data using machine learning models. Additionally, we suggested some initial ideas for this architecture to inspire future research and development in this area. Authentication is distributed among the participating clients. When a new device wants to access the network, it must be verified by a certain number of peer devices. There can be a number of thresholds that accept the device joining the network. Some public key cryptography that is based on grouping, such as Shamir’s secret sharing, can be utilized to authenticate devices. Another approach is to propose a dynamic election mechanism, where a subset of devices elects a trusted cluster head responsible for aggregating authentication decisions and managing secure model updates. This cluster head is chosen based on trust metrics, device reputation, or previous contributions to the network, ensuring a balance between decentralization and efficiency. Figure 8b shows the peer-to-peer architecture.

### 3.3. Challenges Related to Authentication in FL

In this subsection, we investigate the most common challenges and attacks in the field of federated learning authentication. Moreover, we discuss several attacks that may threaten the authentication process in FL networks.

1. Single point of failure: Most FL structures are based on a centralized server. This server performs the aggregation process, as well as authentication and identity management [41]. If this server gets compromised, the whole system will be at risk. The compromised server can cause several problems, outlined as follows: (1) it can expose authentication credentials; (2) generate false or biased general models; and (3) disrupt the entire federated learning process. Several research works solved this problem, either by using blockchain or distributed ledgers, such as [14,41,54,57]. Some authentication mechanisms used hierarchical or peer-to-peer structures to distribute the aggregation process and avoid bottlenecks, such as [39,49].
2. Poisoning attack: This is one of the most common issues related to FL [16]. It is divided into the following two main categories: model and data poisoning. The main target of this attack is to inject either the data or the model with malicious inputs to cause accuracy degradation, a biased model, or a backdoor. An example of data poisoning is that a user may flip labels (labeling spam emails as “safe”), and for model poisoning, an attacker alters model gradients to make it classify inputs incorrectly. This type of attack is severe, especially in a biometric or behavior authentication system in a hospital, where unauthorized users may be classified as legitimate users [43]. Some research concentrates on mitigating only external poisoning attacks, while others also take into account internal threats. For example, Ke et al. [59] proposed an authentication mechanism which embeds a Message Authentication Code (MAC) into encrypted model parameters to ensure the authenticity and integrity of updates from legitimate clients. This model integrates identity authentication, watermark provenance tracking, and homomorphic encryption.
3. Non-IID (Non-Independent and Identically Distributed) data: This problem occurs because different clients may have different data distributions [12]. These differences may cause several issues, such as inconsistent model updates, failure to find an optimal solution or to converge, and a biased model for certain clients or groups. The non-IID problem becomes even more critical in biometric and behavior-based authentication systems, as these data types are highly user-specific and vary significantly between clients [43].
4. Byzantine attack: This refers to malicious clients or Byzantine clients who are trying to gain official access to the network. This type of adversary is intentionally disrupting the collaborative training process by sending incorrect or misleading model updates to the central server. The clients may use poisoning attacks, i.e., flip labels, backdoor triggers, or add noise, to target the global model. However, they are hard to detect as they may register as a legitimate user in the system [60]. Recent work presented by Li et al. [61] focused specifically on mitigating this attack by presenting a tripartite authentication framework called BRFLATA. Before each training, the server uses a credibility table to pair each client with another trusted client, who will act as an authentication party to verify the integrity and correctness of the uploaded model parameters. The authentication process is tripartite because it involves the following three parties: the original client, its pair authenticator, and the server, each carrying out a specific role in verifying the model updates. The aggregation process depends on the credibility of the clients. If the score goes up, it means the authentication is successful and the client’s updates are accepted; otherwise, it is suspected of being Byzantine or attacked.
5. Man-in-the-middle attack (MITM): This type of attack occurs when communication is intercepted between parties. The attacker will try to impersonate one party (client or server) to send malicious model updates. One of the most well-known MITM attacks in authentication systems is session hijacking. The attacker will try to steal the user credentials from a legitimate session between the FL server and the client and try to impersonate the user and send fake updates [9].
6. Secure model updates: This will ensure that only authenticated, legitimate devices contribute to the global FL model while protecting the integrity and confidentiality of model parameters. Some updates may reveal sensitive or private information about users and their identities. As a result, ensuring anonymity and securing gradients in authentication mechanisms is critical [48]. In [62], the authors used Generative Adversarial Networks (GANs) to demonstrate that model aggregation in FL can leak private information about users. Their experimental results revealed that it can operate undetected on the server and exploit model updates to infer private user data.

## 4. Blockchain-Based Authentication

Traditional FL depends on a centralized server for aggregating, collecting, and broadcasting the global model. This architecture can lead to a single point of failure, the threat of a malicious server, or bias toward specific clients [25]. To overcome these challenges, several researchers have integrated blockchain technology with federated learning networks. In the authentication-driven FL, blockchain is being utilized in multiple directions. It can be used as a decentralized system, where clients authenticate through verifiable credentials stored on the blockchain, or the blockchain can be used to aggregate and store the global model [44]. It also provides a tamper-proof log for all authentication events. Additionally, smart contracts can automate access control and ensure that only verified participants contribute to model training. Blockchain enables a consensus mechanism to validate if a client is authorized to participate [45]. In this section, we discuss several recent schemes for authentication in FL using blockchain and compare them against several evaluation criteria and security attacks for authentication in FL. We also classified these authentication schemes based on their blockchain type, since the type influences the trust model, consensus mechanism, and the scalability of the authentication mechanism. Accordingly, these schemes are categorized based on whether they used private or consortium blockchain.

### 4.1. Private Blockchain

This type of blockchain is permissioned and restricted to a specific enterprise, group, or organization. It is less decentralized and less scalable compared to the public type, as it restricts participants and controls the consensus algorithm [26]. The private blockchain has several features, like high throughput, low latency, and controlled access, which makes it suitable for resource-constrained environments, such as IoT, IoMT, etc.

Xiong et al. [63] proposed an authentication scheme based on private blockchain to overcome problems related to a single point of failure. They improved the previous blockchain mechanisms in the following three dimensions: First, it replaces KDC with Shamir’s secret sharing algorithm [64]. Second, it designs new consensus algorithm. Third, it uses differential privacy. This work provides a more decentralized approach than previous works [14,65] by replacing the central server with smart contracts and using Shamir’s secret sharing algorithm [64] instead of depending on KDC. Each node generates its own private-public master key pairs, and then receives partial private keys from other nodes and combines them to compute their final public–private pairs. The authentication process is based on an identity-based revocable ring signature (RRS) scheme, which allows nodes to prove themselves anonymously without revealing their identities. This ring signature allows the revocation authority (blockchain) to reveal the true identity of malicious participants if the node behaves suspiciously. The cryptographic algorithm used here is built over two cyclic groups of prime order with a bilinear map, using collision-resistant hash functions for identity mapping. The security depends on classical hardness assumptions of the discrete logarithm and as a result it is not resistant to quantum attacks. Furthermore, the authors designed a proof of accuracy (PoA) consensus algorithm to reduce the energy consumption and the computation cost. PoA ensures low-quality model updates are filtered out by accepting only model updates that achieve a predefined accuracy threshold on a shared test dataset. This model integrates differential privacy using Gaussian noise to ensure the model’s updates privacy.

DA-FL achieved stable convergence compared to its competitors with only around 3.17% accuracy loss, while mitigating the malicious nodes positioning attack. This model provides a robust decentralization approach; however, there is a computational overhead for the distributed key generation, which gets larger when more nodes join the network. It also suffers from model inversion and side-channel attacks. Moreover, for the autonomous driving environment, the authors in [13] proposed a blockchain authentication scheme. This protocol is presented to solve problems related to centralization approaches, such as depending on KGC or single servers for aggregation. Additionally, it focuses on designing an efficient protocol that overcomes extensive computations. The protocol employs blockchain as a decentralized, immutable ledger for storing authentication records and facilitating cross-domain vehicle authentication. The incorporation of the Delegated Byzantine Fault Tolerance (DBFT) algorithm increases the model’s throughput and provides fault tolerance. It also maintains ledger consistency, making it suitable for dynamic vehicular environments.

The proposed solution enhances the efficiency and security of autonomous driving environments using FL and blockchain. It addresses critical challenges such as privacy, data security, and computational overhead, as it reduces the communication overhead by 40.3% compared to similar approaches. However, it still has some issues to address, as the model depends on idealized hardware environments, and its assumption of a fully trusted authority (TA) raises doubts about real-world applicability and resilience to insider threats. Additionally, the protocol keeps some secrets constant for a long time without changing, like shared keys between TA and vehicles/RSU and vehicles’ anonymous ID. This lacks forward and backward secrecy and can cause several vulnerabilities, like impersonation and tracking vehicles.

Recently, homomorphic encryption (HE) has gained attention in the FL domain due to its ability to securely aggregate masked values without exposing their sensitive contents. Numerous scholarly investigations, notably the contributions by [66,67,68], have integrated homomorphic encryption techniques into FL architectures to ensure privacy and support secure aggregation. Within the specific context of authentication in the FL domain, only a few studies have integrated HE to secure the aggregated gradients. Three significant contributions to this field [14,56,57], are comprehensively examined in this section. Additionally, the approach proposed by Yazdinejad et al. [40], which represents an artificial intelligence-based solution, is subjected to in-depth analysis in Section 6.2. Abdur Rahman et al. [56] proposed an authentication scheme, using permissioned (Ethereum + Hyperledger) blockchain in FL. To authenticate clients, the scheme generates a unique blockchain identity for each device and records every model update as a transaction, which allows verifiable and tamper resistant auditing. The training process consists of local model updating, encrypting the gradients using HE, and submitting these gradients to the blockchain smart contract to be aggregated in the Software Guard Extension (SGX). This method utilizes blockchain and smart contract to ensure that only authenticated devices participate in the global model.

Additionally, it ensures data provenance, tracking what each node contributes to each update. This framework also utilizes differential privacy and homomorphic encryption to preserve privacy and security for both the data and the model. It concentrates on the protection of IoHT devices and healthcare data, especially COVID-19 applications and use cases. For hardware-level security, a trusted execution environment, namely, Intel SGX, is integrated with the blockchain to ensure that even aggregation nodes cannot see or manipulate the raw data or model parameters. Although this framework provides strong privacy protection, it does not clearly mention or clarify the threat model and attack analysis and there is no details about the consensus algorithm for the blockchain [19]. Moreover, it requires heavy computational resources for training, which might be an issue for limited edge nodes’ capabilities [69]. The differential privacy technique that is used in this work may cause data utility loss, and this will effect the availability of the IoHT devices [70].

Another work that implements homomorphic encryption is presented in [57]. Gupta et al. [57] integrated blockchain technology with FL to provide a trust secure authentication mechanism for personal consumer electronics systems. This work also implements HE to preserve the privacy while transferring the model updates. It is build on the following three main layers:

1. The user layer, where the local model is trained and the authentication process is performed based on the following three factors: ID, password, and biometric data.
2. The second layer is the fog local layer, in which the local encrypted models are aggregated.
3. The cloud layer, which performs the global aggregation across fog nodes.

In this scheme, Paillier homomorphic encryption is applied on local model updates before transmission, enabling secure addition at the fog layer without decrypting the individual gradients. The blockchain is implemented to store hashed model updates to ensure tamper resistance and immutability. This model processes in a hierarchical manner to reduce communication overhead between edge devices and the cloud and improve scalability. It structures the learning process into periodic cycles, where each fog node collects encrypted gradients from nearby devices, performs homomorphic accumulation, and forwards the aggregated ciphertext to the cloud for the final model update. While this framework provides decentralization and privacy, it still faces some issues due to computational overhead. There is no evaluation of the latency or energy consumption caused by encryption [71]. In addition, this work does not provide any security analysis or threat model to clarify which attacks are prevented. Although it is using blockchain, it does not explicitly mention any details about the type or the consensus algorithm, which makes it hard to evaluate the throughput, latency, scalability, and energy consumption. Moreover, the authors do not explicitly evaluate or quantify the computational costs of homomorphic encryption in terms of latency, energy consumption, or device performance.

### 4.2. Consortium Blockchain

This type of blockchain is permissioned and governed by a group of organizations. It is similar to the private blockchain in its performance; however, it allows a larger number of participants to join the network [26]. It balances between decentralization and efficiency by allowing multiple trusted stakeholders to validate transactions while restricting public participation. This type enables decentralized authentication, which makes them suitable for collaborative networks, like FL. Moreover, it maintains the operational control and trust boundaries. Ji et al. [45] presented an authentication scheme for IoT called LAFED, which integrates FL with blockchain. This mechanism uses consortium blockchain to address the privacy and efficiency issues associated with traditional FL methods, such as depending on a single trusted server for authentication, or issues related to distributed authentication. This research focuses on the following three main directions: a lightweight authentication, a zero-knowledge proof (ZKP) consensus algorithm, and an aggregation method based on node contribution and model quality. The blockchain is used to replace the central server in FL and to store user registration data, authentication parameters, and model updates. Furthermore, the authors proposed a flexible lightweight consensus method that dynamically selects consensus committee members based on the quality of the local models and data contribution metrics of participating nodes. The nodes are selected based on their contributions and measured using some metrics like cross-entropy and data information entropy. They are prioritized based on their high contribution to ensure that the committee contains only reliable and trusted participants. The committees are responsible for verifying the identity of participants using ZKPs, as well as ensuring the validity of model updates and adding trusted local model parameters securely to the blockchain.

It is noteworthy that the consensus committees are responsible for managing the authentication process for their participants, while the blockchain acts as a secure ledger where key information, user registration details, and public parameters are stored. This process ensures privacy since sensitive information is not revealed and ensures that the authentication process is decentralized and tamper-proof. Moreover, the authors proposed a novel aggregation algorithm to collect the local parameters to produce the global model. This algorithm overcomes problems related to FedAvg, such as producing low-quality global models caused by poor local model training. As it evaluates the quality of local models using a quality evaluation weight that is derived from the model loss during local training. The proposed system achieves high model accuracy, with minimal degradation of performance due to differential privacy noise. Additionally, LAFED’s performance was compared against other models, demonstrating good performance in terms of accuracy and convergence speed. However, there are still some limitations that need to be considered. First, the paper mainly focused on poisoning attack prevention only, without considering other adversarial scenarios, such as evasion attacks or model inversion attacks. Additionally, this model is vulnerable to impersonation, session hijacking and DoS attacks [58]. While it provides secure initial authentication, it lacks continuous monitoring of the model during training and upload processes. This issue makes it vulnerable to model tampering or node defection because the authenticated node may behave maliciously or fail to contribute as expected in the training process [41]. Finally, issues related to data distribution need to be addressed, such as non-IID (non-independent and identically distributed) data, which can degrade the model accuracy while using differential privacy mechanisms.

Another authentication scheme that uses consortium blockchain with FL for a 5G smart Unmanned Aerial Vehicle (UAV) environment is presented in [14]. Feng et al. [14] proposed a secure authentication framework, called BE-DHFL. It is based on horizontal FL and blockchain technologies to eliminate the security and privacy issues related to a centralized server. It also addresses the challenges in cross-domain UAV authentication and data privacy by using smart contracts for both model aggregation and authentication. The system is composed of the following three layers: P2P network, blockchain, and application. The P2P network layer contains committee nodes that maintain the blockchain and execute consensus algorithms. It trains the nodes to perform local updates, and it then secures their model parameters using homomorphic encryption before contributing to the global aggregation process. The blockchain layer serves as a decentralized and secure platform and uses a consortium blockchain to record transactions, including identity management and model updates. The application layer contains the KGC center for drones’ local and global registration as well as identity management.

Furthermore, the model implements the following two types of blockchains: private blockchain that manages resources within the domain and consortium blockchain to authenticate devices and update models across the domains. The data in these blockchains are divided into blocks as follows: authentication blocks that store data related to authentication, and training blocks to record updates from both local and global models. The global model is not updated from the local drone model updates immediately; however, these local model updates are applied to a model pool until it reaches a predefined threshold or the waiting time expires. BE-DHFL ensures that only authorized devices are incorporated into the system by authenticating them. The authors conducted a security analysis, proving that the model is secure against several attacks. The model was also evaluated in an experimental environment, proving that the model achieves an accuracy of around 94% in a short time frame. This makes it convenient for drones because of their resource constraints. However, several limitations are related to this work, such as latency and communication overhead. While training the model, this solution does not take into account malicious behavior [72]. Furthermore, it does not address incentives for participants, which can reduce the number of contributing drones. The dependency on KGC for providing private keys might cause threats to the whole encryption process. There are also vulnerabilities related to the third-party blockchain, such as pooling attacks, collusion attacks, and data tampering [73].

Liu et al. [41] also presented an integration of blockchain with FL to implement a secure authentication mechanism for VANETs. It provides a lightweight continuous mutual authentication protocol using Chebyshev Chaotic Maps to generate session keys that secure the communications between vehicles, fog servers, and cloud servers. The FL process is performed in a hierarchical approach; first vehicles train their local models and send masked, signed updates to fog servers, which perform region aggregations. Then, these fog model updates are sent to cloud servers for global aggregation. Additionally, the blockchain is utilized to manage identity registration, enforce authentication logic, and maintain a tamper-proof reputation and revocation record for the vehicles. This protocol provides a continuous authentication through signature validation, which limits the ability to detect malicious behavior after initial authentication, such as poisoned model updates or compromised but authenticated nodes.

Recently, Ahmed and Anisi [74] presented a post-quantum authentication for FL in Vehicle-to-Grid V2G network. This work utilizes consortium blockchain to store all the certificates and identities of participants, while relying on off-chain to verify the certificates during the authentication. To the best of our knowledge, this is the only post-quantum authentication framework for FL. It also uses the device PUFs to authenticate devices and improve the hardware security. It uses quantum resistant digital signatures, like: Lightweight Merkle Signature (LMS) and eXtended Merkle Signature (XMSS). These signatures are implemented to authenticate vehicle requests, in addition to using them together with the PUFs values to generate session keys. The authentication process is supported for both inter-domain and cross-domain applications with anomaly detection. The authentication process for the inter-domain application is between the vehicles and the nearest charging station using off-chain verification. While in cross-domain authentication, the request is passed to the edge node using ZNP and anomaly detection. This will ensure that the edge node will accept only normal updates coming from the vehicles. However, the security of edge nodes/charge stations’ infrastructure is assumed but not fully addressed.

The authors construct formal/informal security analysis and test their model using NS3. Results showed storage decrease, computational cost reduction, and throughput improvement. The computed metrics only show the detection accuracy using FL, which makes it unclear how the model performs in real-world imbalanced scenarios. This framework has some limitations, outlined as follows:

(1) No backward secrecy, as the renewal of the session key depends on the old key.
(2) PUF reliability is sensitive to the noise environment.
(3) Malicious or compromised edge devices may inject false updates, bias the global model, and reduce anomaly detection accuracy.

All the previous works utilize sequential blockchains; however, Fan et al. [44] proposed an authentication framework based on a Directed Acyclic Graph (DAG) blockchain. It uses DAG and dynamic accumulators to address security and efficiency challenges in FL. The proposed framework reduces the dependencies on centralized servers like Key Generation Centers (KGCs) or the FL aggregation server. It allows participants to generate their own public–private key pairs and manage their identity securely. It also used Elliptic Curve Cryptography (ECC) and hash functions for cryptographic operations, including lightweight digital signatures for authentication. The hash functions serve various purposes as follows: mapping identities to accumulator values, combining pseudonyms with local models for signature creation, and generating pseudonyms to ensure anonymity. The framework consists of four main entities, as follows:

- Trusted Authority (TA): Initializes the system by generating public parameters and assigning pseudonyms to workers, enabling anonymous interactions.
- Task publishers: Responsible for initiating global model requests, as well as verifying users’ signatures, identities, and local models during FL training. It can also perform batch signature verification to reduce the computation overhead, as many workers can submit at the same time.
- Worker nodes: Train local models, independently generate public–private key pairs, and register their pseudonyms and public keys on the blockchain through the accumulator.
- Blockchain: Provides decentralization by using a DAG structure. This allows multiple transactions to be verified in order to improve speed, scalability, and decentralization.

The authentication process starts by registering the worker nodes to the TA through their IDs and public keys to generate their pseudonymous (masked) identity. Each node is responsible for generating its own public–private key pairs, based on the global parameters that are sent from the TA. The TA is the only party that can track each user and recover their real identity, which helps in preventing malicious users. After that, during the FL process, worker nodes sign their local models using their private keys together with their pseudonyms, and they submit them to the blockchain committee along with proof of authenticity. Additionally, if the transaction is validated by the committee, the task publisher can collect these verified models from the blockchain and apply aggregation methods.

The authors performed a security analysis for the model. They also conducted an experimental test, which demonstrated that DAFL achieves lower authentication costs, enhanced accuracy in the presence of malicious workers, and improved overall scalability compared to traditional centralized schemes. This research provides a decentralized and lightweight anonymous authentication scheme for FL using blockchain and dynamic accumulators to address security and efficiency challenges. This accumulation process is used to compress identity records, which provides less computation overhead and can be quickly verified using the witness proof. While the proposed scheme effectively eliminates centralized reliance and enhances efficiency through batch verification, it has some limitations. The system lacks robust accountability mechanisms for malicious participants and struggles to balance anonymity with traceability, as pseudo-identities for each client does not satisfy unlinkability [75]. It does not incorporate data minimization or strong privacy protection [76]. Additionally, local model parameters are transmitted in plaintext, exposing them to potential attacks, such as poisoning and eavesdropping [75]. Despite improving computational overhead and reducing storage requirements, it overlooks critical aspects such as compliance with privacy standards and real-time monitoring, which are essential for practical and secure FL deployments [76]. Table 1, provides a summary of all the blockchain authentication mechanisms in FL. It provides the main contribution for each scheme, the authentication type, blockchain type, and consensus algorithm, performance metrics. Finally, it highlights the advantages and limitations of each scheme. In order to provide a clear overview, the table summarizes the essential characteristics of each blockchain-based authentication mechanism in FL.

To further clarify the computational characteristics of the surveyed schemes, we present a unified complexity analysis table. Table 2 contains all the cryptographic operations used during both registration and authentication phases at all of the following three layers: client, edge, and server. The table abstracts the computation into generic cost units (e.g., bilinear pairing, homomorphic encryption, and digital signature), which enables comparison without being related to specific platforms or runtime boundaries. Note that XOR and concatenation costs are ignored as negligible due to their minimal computational impact. All symbols related to the computation cost analysis can be found in Table 3.

### 4.3. Comparing Blockchain-Based Authentication Techniques

In this subsection, we compared blockchain-based authentication schemes, based on a set of defined evaluation criteria. These criteria are derived from several key security requirements and common attacks that each authentication scheme is expected to address. These criteria are divided into the following two categories: (1) security requirements that the protocol must satisfy, and (2) types of attacks that the protocol is designed to prevent. These criteria are defined as follows:

**First: Security requirements**

- Mutual authentication: It refers to when both participants are authenticating each other.
- Continuous authentication: It continuously verifies the legitimacy of users/devices during an active session, rather than relying on a single initial authentication step. Notice that providing continuous authentication can prevent Byzantine clients and session hijacking.
- Anonymity: It is the ability of the authentication scheme to secure the identity of users/devices involved in communication, ensuring that they remain anonymous to unauthorized entities.
- Traceability: It is the ability to track and identify malicious or misbehaving users in case of an attack or violation. TA or blockchain keeps track of the identity and users.
- Perfect forward secrecy: It ensures that even if a long-term cryptographic key is compromised, all previously established session keys will also be secure and cannot be decrypted retroactively.
- Backward Secrecy: It ensures that if a session key is compromised, it will not cause any threat to the security of future communications or keys.
- Secure aggregation: It means preserving the privacy of the model’s parameter, using masking techniques like differential privacy, homomorphic encryption, etc. This will ensure the privacy of the parameters, even in the aggregation process.
- Decentralization: If the protocol depends on Trusted Authority (TA), in authentication or identity, it will be considered as centralized; otherwise, it will be decentralized.

**Second: Security attacks**

- Resistant to MITM: Whether the protocol is preventing man-in-the-middle attacks.
- Resistant to poisoning attack: Whether the protocol is preventing poisoning attacks.
- Resistant to impersonation attack: If the protocol prevents one participant from masquerading and pretending to be someone else.
- Resistant to quantum attacks: If the protocol can prevent future quantum attacks, by using post-quantum algorithms.

Table 4 illustrates the differences between each mechanism against the predefined evaluation criteria. If the protocol achieves the security aspect, then it is marked with (✓); otherwise, it is (×) or (NA), which means that this security aspect is not applicable to the protocol.

## 5. Cryptography-Based Authentication

Recently, several research studies have concentrated on utilizing cryptography to authenticate and secure devices in federated learning. Public-key cryptography has been widely used in IoT authentication in FL. It is used to transfer model updates securely, protect clients’ identity, and prevent adversarial attacks [55]. Recent improvements in FL security focus on secure aggregation, leveraging several cryptographic techniques like homomorphic encryption and secure multi-party computation. These methods can aggregate the encrypted data, without the need to reveal them. In this section, we explore several recent authentication schemes that utilize lightweight cryptographic algorithms, such as Elliptic Curve Cryptography, digital signatures, zero-knowledge proof, and hash functions.

In addition, we examine some lightweight computations like XOR (exclusive OR) and concatenation. We observed that most schemes implement ECC in their models, due to its ability to provide high security with smaller key sizes, which makes it suitable for resource-constrained IoT devices [78]. We classified this cryptography-based authentication area into the following two main branches: Public Key Infrastructure (PKI)-based and certificateless schemes. PKI-based approaches rely on a trusted certificate authority (CA) to issue and manage digital certificates for each client, ensuring identity authenticity through signed credentials. On the other hand, certificateless schemes eliminate the need for certificates, thereby reducing overhead and simplifying key management—making them particularly appealing for large-scale, resource-constrained IoT deployments. We reviewed each approach in detail, presenting the schemes’ contributions, problem definitions, and results. Finally, these schemes are compared against the evaluation criteria defined for authentication in FL, which are categorized into security requirements and attacks prevented.

### 5.1. PKI-Based Authentication Schemes

Deebak and Hwang [39] presented an authentication framework called L2FAK for IoMT. This framework utilizes lightweight cryptography and FL to ensure secure and efficient authentication. It is based on two-factor authentication, using possession-based factors (secret keys and temporary pseudo-identities) and knowledge-based (password and ID). The system focuses on a layered architecture using fog and cloud computing for efficient data handling and reduced latency. The FFLA algorithm is to ensure that only legitimate devices can access the network and any deviation or new attributes that occur will require further investigation. L2FAK works in the following two ways: first, it secures and authenticates users joining the network using cryptography. Then, it utilizes FL to apply an extra layer of authentication. However, the model was tested using non-medical datasets, despite its focus on IoMT [2]. This could limit the framework’s applicability and performance in real IoMT scenarios. It lacks in addressing some IoMT attacks, like spoofing, alteration attacks, and traffic attacks, such as manipulating data flow patterns or sensor anomalies [79]. This protocol is using a long-term secret key that is not changed and is assumed to be stored securely in both gateway and device, but if this key gets compromised, attackers can impersonate the user and generate a future session key. This is a critical point that needs to be addressed, especially since we are dealing with crucial medical devices. FLLA concentrated only on devices’ attributes or parameters (secret key, session identity) for authentication rather than behavioral data. This can be applied as an area of improvement by adding some devices/users’ behaviors to enhance the detection of compromised or malicious devices.

Another research implemented ECC for continuous authentication in FL is presented in [42]. It is a continuous authentication scheme for federated learning in VANETs. FL has been widely adopted in vehicle networks, allowing devices to train the global model while keeping their data localized. However, these existing FL systems face challenges such as malicious clients injecting poisoned data and privacy vulnerabilities. Additionally, current aggregation protocols lack continuous monitoring to block malicious clients during the training process.

The model used an aggregation architecture that contains a two-phase process using Edge Devices for preliminary validation and RSUs for final aggregation. Moreover, the TA is responsible for issuing the secret parameter, dividing the network into domains, where each is managed by a specified RSU and can reveal any device’s identity to prevent malicious users. The authentication process starts with clients and RSUs registering and exchanging their public keys to establish secure communication via Elliptic Curve Diffie–Hellman (ECDH). Clients encrypt their masked gradients using session keys and group signatures, which are then verified and authenticated by the ED and RSU before aggregation and unmasking. Notice that the continuous authentication process is carried out by the ED and RSU. The ED verifies each client using their signature, while the RSU verifies the ED through its signature and by evaluating the received parameter. If any parameters are suspicious, RSU will contact the TA for confirmation. To preserve anonymity, the model incorporates a modified group signature scheme, enabling vehicles to sign data while ensuring unlinkability and resistance to identity exposure. The system was tested on common datasets like MNIST and CIFAR10, showing lower computational and communication costs compared to other schemes. The authors also conducted an experiment on real VANET applications, using some real-world traffic. This research introduces a continuous authentication scheme that uses FL and integrates cryptographic algorithms. It employs non-interactive zero-knowledge proof (NIZKP) to validate the authenticity of participating clients without revealing sensitive data. Results showed that the proposed scheme achieved faster training convergence, reduced communication overhead, and improved accuracy. This research presented an innovative approach to address privacy and security issues related to FL in VANETs, taking advantage of cryptographic techniques such as zero-knowledge proofs, gradient masking, and group signatures. It depends on applying continuous authentication using a two-phase aggregation scheme, which reduces computational overhead. However, it contains some limitations, outlined as follows:

- This scheme fails to address both the revocability and anonymity during the authentication process. As a result, it may be vulnerable to exposing the identity information of edge nodes, potentially compromising privacy and leaving the system vulnerable to misuse or unauthorized access [80].
- The actual bandwidth requirements for transmitting masked gradients, verification messages, and the authentication process in high-traffic scenarios could be a challenge [42].
- If the TA is compromised or becomes a bottleneck, the entire security model could be at risk, especially because it holds all the IDs and the verification process.
- Even though there is continuous authentication, an authenticated client could still submit poisoned gradients, leading to model corruption over time.
- Moreover, the research did not discuss non-IID, which is a common and important issue in VANET.

### 5.2. Certificateless-Based Authentication Schemes

Certificateless cryptography is an alternative cryptographic method used to ensure secure communication without depending on digital certificates. Unlike traditional public key infrastructures, it reduces dependence on third-party authorities by leveraging mathematical algorithms to establish trust. Certificateless cryptography divides the private key between the user and the authority, which reduces the total control of a single authority and mitigates the risks associated with key escrow [81]. Additionally, some schemes use identity-based signatures, which derive public keys directly from users’ identities. This will eliminate the need for PKI and digital certificates. Several FL authentication mechanisms use this certificateless approach to avoid problems related to certificate management.

Li et al. [46] proposed a certificateless authentication framework for trustworthy 6G communications called CATFL. The framework concentrates on securing the server to prevent model poisoning as well as on securing the clients while preserving privacy. Mutual authentication between client and server is performed to ensure their identities. Also, both client and server must verify signature information to validate the model updates from each party. It is based on certificateless cryptography to avoid the management overhead of certificates in PKI systems. CATFL achieves anonymity and conditional traceability using pseudonym generation. The authentication process consists of several steps, starting with a setup phase that initializes the system parameters by KGC and TRA. Then an anonymous ID is assigned to each client and server, as well as the partial private key. Next, every entity generates its full private key by integrating the partial private key with a locally generated secret value. Using this key pair, entities can securely sign and verify messages. During the signature phase, clients sign their model updates, and the server signs the global model before distribution. At the end, the authors provide a theoretical security analysis and prove the robustness of the presented framework. CATFL provides a dual verification mechanism, ensuring the integrity and trustworthiness of the federated learning process, while mitigating risks such as model poisoning and unauthorized data manipulation. It also overcomes problems related to certificate management in PKI systems. However, all the capabilities to trace real identities from pseudonyms are held by the TRA, which raises concerns about privacy, misuse, or attacks on this server. The model relies on KGC and TRA can cause scalability issues and suffer from a single point of failure. CATFL concentrates mainly on poisoning attacks, while other threats are not explicitly considered in the framework, like inference attacks, model inversion or side-channel attacks. Moreover, the authors only provide theoretical proof for the framework, this limits the ability to check the performance of the system under varying network conditions and attack scenarios.

Another approach that uses certificateless is presented by Huang et al. [47]. The authors proposed an authentication scheme for Industrial IoT (IIoT) in FL, called EPPAFL. It is based on certificateless, lightweight cryptography to overcome problems related to resource-intensive algorithms. This protocol balances data usability with privacy protection by combining authentication, anonymity, verification, and privacy protection. The authors improved efficiency and performance by avoiding computationally intensive mechanisms, such as homomorphic encryption, and instead applied ECC-based certificateless signatures to secure the updated weights. The server utilizes batch verification using group key, which is managed by the Chinese Remainder Theorem (CRT). The server also uses TESLA (Timed Efficient Stream Loss-tolerant Authentication Protocol), which is a lightweight broadcast authentication protocol depending on delayed key disclosure and hash chains. The protocol provides several security aspects as it provides authentication, integrity, anonymity, untraceability, and privacy. It also ensures non-repudiation and backward/forward secrecy while preserving privacy. This protocol provides a lightweight computation for IIoT devices, which improves their efficiency and performance. However, several limitations exist that leave space for further improvements, such as the following:

- It prevents poising attacks from external users that are not authenticated; however, legitimate participants can send malicious/misleading updates.
- If the cloud server is not fully trusted or compromised, model parameters can still leak some private information.
- If the TA is compromised, all the users can be traced, so it is not fully untraceable.
- No encryption is performed, which means the parameters may leak some confidential data.

Moreover, Yuan et al. [48] presented FedComm, which is a certificateless authentication protocol to preserve privacy for federated learning in VANET. It addresses privacy, efficiency, and security, which are common challenges in VANETs, and ensures secure collaboration among vehicles while maintaining the anonymity of participants. It concentrates on implementing unlinkable pseudonyms to anonymize vehicles, preventing attackers from correlating local model parameters with real identities. This protocol is based on certificateless cryptography to generate vehicles’ public and private key, without the need for digital certificates. This simplifies the key management part and presents a cost-effective solution. All the encryption and signatures are performed through ECC. Additionally, the Chinese Reminder Theorem is used to help in defining and managing communication groups among vehicles dynamically, which will perform batch verification.

This protocol presents a considerable effort to enhance privacy and authentication in FL for VANET, addressing critical challenges related to VANET. However, the protocol suffers from several limitations, such as vulnerabilities to signature forgery and traceability failures, which undermine its claimed security features [82]. Additionally, its reliance on a centralized TA introduces a single point of failure, and practical issues like scalability, dynamic key management, and communication overhead in highly dynamic VANET environments are insufficiently addressed. While the theoretical contributions of the paper are significant, these limitations need to be resolved for the protocol to be practical and effective in real-world VANET. The group generation and updates may become computationally intensive, especially if vehicles frequently join or leave the network. As part of future work, the authors plan to enhance FedComm by incorporating secure and verifiable mechanisms for weighted average aggregation in federated learning, ensuring greater reliability and security in the aggregation process.

Furthermore, Liu et al. [55] presented a certificateless authentication scheme for IoMT. This research mainly concentrates on preserving privacy in FL for IoMT devices using blind masking and re-encryption techniques. It utilizes authentication using certificateless digital signature to ensure that only authenticated clients participate in the aggregation process. This architecture contains a Noise Parameter Server (NPS), which creates noise to mask client’s local models. Additionally, the aggregation server acts as a re-encryption proxy that aggregates and forwards the global model, without revealing the actual data. The overall workflow is structured into three phases as follows: initialization, masked model uploading, and re-encrypted aggregation. This clarifies how the NPS, KGC, and Trace Authority (TRA) coordinate across each FL round. In this process, blind signature issuance and noise vector generation occur before upload, while proxy re-encryption ensures unlinkability between local updates and device identities. Additionally, trusted authorities such as the TRA and KGC provide pseudonym identities and partial public/private key pairs, helping support anonymity and secure authentication without relying on conventional certificates. This scheme provides fault tolerance through choosing another online client and reassigning the noise vectors of the dropped client using re-encryption, without effecting the correctness of the global model. Although the paper describes the sequence of cryptographic operations in detail, it does not evaluate their computational impact on IoMT devices, leaving uncertainties about scalability, latency, and energy consumption. Moreover, this scheme is vulnerable to quantum attacks and no defense mechanisms are included for poisoning attacks.

Wang et al. [50] presented a secure FL authentication for Electric Vehicle Infrastructures (EVIs). It provides a mutual authentication between Charging Stations (CSs) and the Charging Station Provider (CSP) to ensure the security and integrity of local model updates used for energy demand prediction. In terms of methodology, the scheme introduces an identity-based pairing signature system where the KGC generates private keys for both CSs and the CSP. Additionally, the mutual authentication occurs through a two-way challenge/response protocol, and multiple CS signatures are verified simultaneously using batch authentication to reduce overhead. After authentication, session keys are negotiated and used to symmetrically encrypt model updates via AES. As a result, batch authentication, trust-based verification, and signature aggregation overcome common problems related to FL, like data poisoning, model poisoning, free-riding, and information-exploitation attacks. It supports the cross-silo approach as each CS acts as a trusted silo for its local data. This protocol authenticates the participating devices; however, it does not hide the real identity of participants and also suffers from a single point of failure, since it depends on the KGC for authentication [44]. It is also vulnerable to DoS and impersonation attacks [58].

Finally, Table 5 provides a summary of all the cryptography schemes. It provides the main contributions for each scheme, the type of authentication, and the cryptographic algorithms used in this scheme. Additionally, it clarifies the trusted entities used in the system, which provides critical functions such as key generation, identity management, or aggregation. Finally, this table highlights the advantages and limitations for each authentication scheme.

### 5.3. Comparing Cryptography-Based Authentication Techniques

In this subsection, we compared the cryptography-based authentication schemes using the same evaluation criteria previously applied to blockchain-based schemes in Section 4.3. This ensures consistency across both categories, since the security requirements (e.g., mutual authentication, traceability, and secrecy) and resistance to common attacks (e.g., MITM, impersonation, and poisoning) are relevant in both whether authentication is implementing blockchain or only using cryptographic algorithms.

Table 6 illustrates the differences between each mechanism against the predefined evaluation criteria. If the protocol achieves the security aspect, then it is marked with (✓); otherwise, it is (×) or (NA), which means that this security aspect is not applicable to the protocol.

## 6. Artificial Intelligence (AI-Based) Authentication

In this section, we discussed another authentication approach that utilizes FL to improve authentication accuracy for users. This approach depends on machine/deep learning algorithms using behavior and biometric data to authenticate users. This section is divided into the following two directions: biometric and behavior. In each subsection, studies are discussed in detail, presenting their contributions, problem definitions, results, etc. Finally, these reviewed schemes are compared against defined evaluation criteria, which are categorized into the security requirements each scheme needs to satisfy and performance metrics.

In this approach the proposed schemes utilize FL in order to build up an accurate authentication system using biometric or behavior data. To train such systems, large datasets are needed to improve their accuracy. However, transferring and sharing these data face several problems, as it violates privacy and it also requires high communication costs for transferring. Additionally, some privacy regulations, like General Data Protection Regulation (GDPR), impose strict restrictions on data sharing to protect user privacy [16]. Consequently, data island or isolated silos problems emerged, limiting access to diverse datasets and decreasing model training accuracy. The aforementioned problems can be solved using FL, which has been recently integrated into behavior and biometric authentication systems. This approach will ensure privacy and enhance the scalability and adaptability of authentication systems. In this section, we discussed some authentication techniques (static and continuous) that leveraged FL with biometric and behavior features to improve the accuracy of the system.

### 6.1. Behavior-Based Authentication

This type of authentication relies on analyzing patterns in user interactions or activities, such as typing speed, mouse movements, or device usage habits. These methods aim to continuously verify user legitimacy by identifying unique behavioral actions. They are particularly effective in scenarios where traditional authentication mechanisms may fall short [83]. It can also be used to ensure the legitimacy of users during the session through continuous authentication. By leveraging FL techniques, behavior-based systems can improve authentication accuracy, generalization, and security while preserving privacy. Wazzeh et al. [9] presented a continuous authentication scheme for a Mobile Crowdsourcing (MCS) environment, using FL to authenticate users based on their behaviors. The model overcomes problems related to continuous authentication using FL, such as non-IID data issues, model divergence, and privacy, while verifying users continuously during the session. It integrates transfer learning and warmup techniques to enhance the accuracy rate. Transfer learning will reuse or fine-tune another model for a different but related task to avoid training the model from scratch. This can improve the authentication accuracy, solve non-IID problems and there is no need for large dataset training. By integrating warmup and transfer learning techniques, the model mitigated problems related to weight and model divergence. It also improves the authentication accuracy, while using aggregation with the FedAvg algorithm.

Additionally, initializing the model with pre-trained weights allows the authentication framework to adapt to user-specific behavioral patterns quickly and minimize the computation overhead. The model suggested sufficient improvements in handling the diversity of users’ behaviors used for authentication. However, there are some limitations that can be addressed, such as the following:

- The research concentrates on solving some problems related to FL diversity, but it has not discussed the security attacks that are commonly related to FL, like model/user poisoning or adversarial attacks. Especially since this paper is related to a security aspect, which is authentication.
- High-dimensionality data like images have slow convergence.
- High communication costs.
- While the warmup approach improves generalization, FedAvg still aggregates a single global model for all users. This might not be ideal for highly diverse behaviors.
- More performance metrics need to be considered.

A similar work is presented by the same author [52]. The main contribution is applying the warmup phase to improve the performance by using a small amount of the users’ data that are sent in the first training round to initialize a solid base. It also utilizes transfer learning for feature extraction to overcome the non-IID problems. This mechanism provides faster convergence; however, the small amount of data shared with the server might contain sensitive or private data. Moreover, it suffers from biased device selection, as some clients may be selected more than others, which can cause a suboptimal global model [84].

Additionally, Cheng et al. [43] proposed a privacy-preserving authentication framework for Virtual Reality (VR) users, utilizing multi-model movement with FL. MetaFL addresses the issues related to traditional authentication in VR, like passwords and PINs, which are vulnerable to attacks like shoulder surfing and guessing. This framework utilizes behavioral biometric data, such as head, gaze, hand, and controller movement to authenticate VR users. The authors considered several fundamental challenges related to VR authentication, such as that users can only access their own motion data, making it difficult to learn users’ variations and recognize unique features, which degrades the authentication accuracy. To overcome these problems, MetaFL effectively selects the appropriate modalities for each user based on their density. The system is designed to cover the followign three main approaches in its development: (1) how to choose the best modalities for every user; (2) how far the data features are from each other when selecting the best combinations for each user to prevent misclassifications; and (3) how to improve model convergence and personalization. While this scheme provides a solution for the VR environment, it still faces some issues as it is vulnerable to other attacks, such as replay or synthesis attacks. It may leak some important information about users’ behaviors while sending the value of the highest density and the average of the embedding vector.

On the other hand, some mechanisms provide device-to-device authentication, such as that in the research of Wehbi et al. [85]. This framework provides a mutual authentication between the clients and federated servers based on trustworthiness. It verifies both parties as follows: clients are assessed through resource usage patterns (CPU, bandwidth, and RAM), while servers are evaluated using collaborative trust bootstrapping and recommendation mechanisms.

In addition, it provides a client selection mechanism based on game theory, in which both clients and servers generate preference lists and are matched through a stable mutual selection process. All previous works usually concentrate on client-side trust scoring; however, in this solution it provides trust scoring computations for both sides. It has been evaluated using MNIST and Fashion-MNIST datasets in non-IID settings to show the improved accuracy and robustness against untrustworthy participants. However, this framework still has some issues, like its vulnerability to impersonation or collusion attacks, where multiple malicious clients can recommend a malicious server. It also may face the cold-start problem in trust bootstrapping when newly joined clients or servers lack sufficient historical data. Additionally, the generic datasets used cannot reflect the complexity of real-world scenarios in smart cities.

Furthermore, Fereidouni et al. [54] provided a risk-based authentication mechanism that uses FL to preserve the privacy and addresses problems related to server-centric approach. This FL authentication scheme operates under a cross-device setting with centralized global aggregation. It implements risk-based authentication that allows/denies users based on risk assessment; this includes checking the user’s history, the current authentication context, and the sensitivity of the assets or resources being accessed. The risk assessment is implemented both at the login phase and continuously during the session using an event-driven process. To overcome the non-IID problem and reduce the impact of heterogeneous user data, this scheme utilizes a special feature engineering based on similarity, which compares how similar current behavior is to past behavior. Additionally, decentralized storage (IPFS) and distributed ledger technology (DLT) are used to enable cross-device learning and the synchronization of the user profile. It also integrates the personalized thresholds with the global model for new users to avoid the cold-start issue. On the other hand, this scheme still suffers from fairness issues, as some users’ behaviors may dominate or bias the global model [86]. It is also vulnerable to model poising attack [86]. The preservation of privacy for private/hybrid clouds works well, but it faces issues in performance and privacy within public clouds [86].

All the previous solutions are used only for user authentication. Alternatively, the scheme of Wazzeh et al. [10] is used for client selection optimization. In [10], the authors proposed a continuous authentication framework called CRSFL that selects appropriate clients for training, while authenticating users. It is based on federated and split learning and addresses the challenges related to clients’ selection during split FL. It takes into account the resource capacities of devices participating in the training process. Split learning (SL) is a distributed ML approach like FL; however, instead of depending on clients only in training the model, SL divides the training of a neural network model between the client and server. This model integrates both approaches to combine benefits from both models. The authors also proposed a clustering approach that groups devices with similar capabilities to improve the system’s efficiency and reduce the communication overhead. CRSFL depends on selecting training clients that have enough resource capabilities to implement. In order to enhance the client selection during the training process, a multi-objective optimization approach is integrated with this model. This optimization uses a Genetic Algorithm (GA) to prioritize clients based on multiple factors, including their resource capacities, data sample sizes, processing capabilities, and unique labels. The experiments of the model were performed using a modular federated learning framework [87] and Pytorch. It used a face detection dataset [88], which contains various face images. CRSFL achieved a competitive accuracy of 89%, comparable to centralized training’s 90%, but with significantly reduced resource consumption and privacy. The integration of SL and FL provides significant advancement in the field of authentication. However, this model has some limitations that can be improved, such as the following:

- The multi-objective optimization problem is NP-hard, which means that as the number of clients increases, the time required to find an optimal solution may become impractical.
- It does not provide secure aggregation, which may leak sensitive data.
- The filtering process may exclude some valuable clients in training because they are resource-constrained.
- More performance metrics in the experiment part need to be considered, as well as improve accuracy.

### 6.2. Biometric-Based Authentication

This type of authentication relies on unique physical characteristics of individuals in order to verify users’ identity. These characteristics are usually biological and difficult to replicate; this provides a reliable and secure form of authentication [89]. Recently, some novel biometrics have become attractive research directions for authenticating users [90], such as finger veins, electrocardiograms, gait recognition, ear recognition, palm vein, and photoplethysmography. Notice that all these new biometrics can verify users without the need for interaction like traditional fingerprint or voice recognition. One of these authentication mechanisms was presented in [15]. The authors implement the Finger Vein (FV) biometric for authentication, called FedFV. FV is an emerging biometric modality that has attracted considerable attention recently. FVs are found under the skin and can only be captured with near-infrared light and a corresponding camera. This approach uses FL to enable decentralized training across multiple clients while preserving user privacy. This model overcomes problems related to non-IID by introducing a novel algorithm called the Federated Weighted Proportional Reduction (FedWPR), which enhances the personalization of the FL models by effectively handling data among different users. The proposed framework divides the client model into shared and personalized components, with shared parts aggregated on the server and personalized parts retained locally. This design is called partial federated learning, which ensures the preservation of client-specific knowledge while still benefiting from the shared learning of federated participants. There are some limitations of this scheme, such as the simplicity of the model personalization, which may result in suboptimal performance for clients with highly unique or diverse data characteristics [91].

Another challenge is the computational burden associated with maintaining N personalized models on the server, which may become increasingly resource-intensive as the number of clients grows. Like other traditional federated learning systems, FedFV introduces significant waiting times during the registration phase, particularly when handling large-scale client registrations [91]. Although this delay is limited to the initial setup and does not affect real-time authentication, it could be optimized to improve scalability and usability. Finally, FedFV struggles with strong heterogeneity of finger vein datasets, lacking an optimal solution for extreme variations between clients [92]. Another work that utilizes users’ biometric features was presented in [12]. Coelhoa et al. [12] proposed a novel authentication scheme for the Internet of Healthcare Things (IoHT), using multimodal biometrics and FL. It combines both Electrocardiogram (ECG) and Photoplethysmography (PPG) signals for authentication, leveraging the decentralized nature of FL to enhance data security and privacy. The benefits of the ECG and PPG are that they do not require specific interaction with the devices, as well as uniquely identifying users. This work implements two cascading Convolutional Neural Networks (CNNs) to train the model locally using PPG and ECG signals. Using two separate CNNs helps the model focus on the unique aspects before combining them, which makes the system secure and robust against spoofing. After training each device locally, the updated parameters are sent to the server, which aggregates by taking a weighted average of the parameters from each device using FedAvg. The authors tested the scheme using the available PPG and ECG datasets, which showed a high accuracy of 99.27% and a low False Acceptance Rate (FAR) of 0.12%. The combination of ECG and PPG signals has enhanced security by reducing the likelihood of forgery, while the FL ensured privacy and scalability across decentralized devices.

This work presents a user-friendly authentication mechanism that allows users to authenticate using several biometric features without interaction. However, there are some limitations in this model. IoHT wearable devices have limited computational battery and power, and training complex CNNs locally could be resource intensive [51]. Additionally, FedAvg is simple and effective, but it may not be optimal for non-IID (non-independent and identically distributed) data, which is common in IoHT scenarios. This could lead to suboptimal model convergence or bias in the global model [16]. Notice that data in FL are non-IID, because user devices generate data from different distributions and each user has his/her unique habits, preferences, behaviors, and biometrics. As a result, the amount of data collected by different clients can vary greatly, causing some problems, such as model convergence, bias in aggregation, and overfitting to local models. Another research utilizes users’ biometrics is presented by Lu et al. [65]. This scheme’s primary contribution is to address the class imbalance for wind turbine fault diagnosis using a privacy-preserving FL framework with gradient noise mechanisms and self-monitoring. It uses facial biometrics to authenticate operators working on the wind turbines. Although biometric authentication is one of the common integrated approach, it does not fully protect against spoofing attacks or insider threats. Several works utilize users’ biometrics, such as Ref. [93], which authenticates mobile users through face recognition by implementing FL and split learning. Also, ref. [94] authenticate users through their voice. However, these schemes are vulnerable to model poisoning and poor generalization [10].

In the past two decades, Internet of Drones (IoD) has become a broad area of research, especially in ensuring security and privacy for these devices. Several techniques are used to authenticate and detect drones, for example, utilizing radar, sound, vision, and Radio Frequency (RF). Recently, RF has been used in detection to overcome multiple problems related to weather, noise, and light that may affect vision and sound mechanisms [95]. Yazdinejad et al. [40] proposed a federated learning model to authenticate drones. This scheme is proposed to overcome the problems related to traditional centralized machine learning models. Unlike centralized models, FL enables decentralized training on local drone data, preserving privacy by keeping data on the device and only sharing model updates with the central server. The model uses Deep Neural Networks (DNNs) and Radio Frequency (RF) features, such as subcarrier spacing and signal power, for drone authentication. Each drone is trained locally and then it sends its updated parameters to the server.

Additionally, the data between devices are sent securely using homomorphic encryption and secure aggregation; however, the authors did not give any details about the homomorphic encryption algorithm.

This framework utilizes drones’ RF biometrics to authenticate devices using FL without sharing any sensitive data. However, this model still encounters some issues, such as data/model poising and Byzantine attacks [17]. Furthermore, the computation of FL-DNN and homomorphic encryption may be overhead, especially for drones with limited capabilities [96]. There are also problems related to RF spoofing and signal interference that have not been discussed. For further improvements to the model, several features from videos and images can be applied to the authentication process. As well as, using these behaviors to continuously authenticate devices during the session.

Another research in the same domain is presented by Zhang et al. [97]. This research utilizes the spectrogram of UAV devices to authenticate or reject the device. It overcomes problems related to traditional authentication and non-contact detection mechanisms. This framework implements the zero-trust concept by authenticating and verifying devices before accepting their participation. It also covers the security over multiple layers as follows: network, data, and task layers. A central implementation of this framework is the Lightweight Spectrogram Network (LSNet), which is a novel CNN used to authenticate UAVs. Unlike existing heavy models such as ResNet or VGG, LSNet introduces Multi-Channel Attention Convolution (MCAC) blocks, which enable the network to focus on time–frequency features in noisy spectrograms with a lightweight size of 1.6 MB only. Additionally, the Class Anchor Loss function was proposed to classify the unknown UAVs accurately. It pulls features of known UAVs closer to their class anchors, while pushing unknown UAVs away from all anchors. Results show an accuracy of 80% for known UAVs, while for unknown UAVs, the AUROC is 0.7. This research overcomes several problems related to the mobility and noisy domain; however, there are some limitations that can be considered to improve the model, outlined as follows:

- Similar UAV types (from the same manufacturer) are hard to distinguish, e.g., (e.g., Futaba T14 vs. T7) produce very similar RF signatures, leading to misclassifications.
- ZT principles, such as authentication, verification, and encryption are included; the research does not deeply detail the cryptographic or protocol-level implementation.
- Experiments used up to five clients, and the model is not tested for its scalability.

Another work that used devices’ attributes for authentication is presented by Istiaque Ahmed et al. [53]. The proposed framework integrates FL and dynamic trust evaluation based on physical layer attributes to provide authentication and authorization for IoT devices. This system continuously monitors the communicating devices by checking several physical layer features, like battery level, link quality, MAC address, Received Signal Strength Indicator, etc. These features are used to compute a trust score to authenticate devices. The system is validated using real-world data collected from Zigbee Zolertia Z1 nodes, which were deployed under several conditions to capture realistic physical behavior. This model was tested over stationary nodes, which limits their ability to work in dynamic or highly mobile environments. Additionally, the authors do not address challenges arising from naturally noisy environments that can affect these physical metrics, such as interference, multi-path effects, temperature drift, etc. Furthermore, Zhang et al. [51] presented a cross-domain authentication for IoT devices (IoTD) called FedScatter. This authentication mechanism operates using a cross-silo FL setting, in which each IoT cluster (or domain) trains a local authentication model using backscatter identity signatures. Each edge server is responsible for collecting these signatures from its local devices. Then the edge server trains the local model for this cluster to send the updated parameters to the aggregated server. This model targets IoT devices and provides a lightweight authentication by utilizing backscatter tags to reflect signals and generate a unique signature for each device. These reflected device-specific features can be seen as unique biometric data for each device. FedScatter introduces a novel parameter aggregation algorithm that assigns weights to local models based on the number of backscatter tags rather than data volume. This algorithm ensures preventing from model poisoning and limits the influence of adversarial clusters. However, it does not prevent model inversion or eavesdropping attacks. It is also a one-way authentication from the IoTD to the edge server, which can be vulnerable to impersonation or spoofing attacks if the receiver gets compromised. This scheme depends on physical layer features for devices with limited resources, but it requires attaching special backscatter tags to each device [98].

Finally, Table 7 provides a summary of the AI-based (behavior/biometric) authentication schemes that are reviewed in this section. It provides a summary of their main contributions, type of authentication, performance metrics used, domain, and the advantages and limitations for each scheme.

### 6.3. Comparing AI-Based Authentication Techniques

In this subsection, we compared the AI-based authentication schemes, including both behavior and biometric. This comparison is conducted according to some defined criteria that include both security requirements and performance metrics. To compare these schemes, we define the following:

**First: Security requirements**

- Continuous authentication: It indicates if the research checks the legitimacy of a user or device throughout the session by analyzing ongoing interactions, such as behavioral patterns or physiological signals, rather than relying on static one-time authentication.
- Non-IID Data Handling: It checks how well the suggested FL model performs under heterogeneous data distributions.
- Personalized model: It indicates if the authentication model adapts to individual users or devices in FL. This metric reduces the errors of the authentication process for non-IID data. To clarify personalized FL, there are three types of personalized federated learning. The first is a single-model approach, where a global model is trained by aggregating client models and then fine-tuned locally for each client. The second is a multi-model approach, where multiple global models are trained by clustering clients with similar data distributions. Finally, there is the N-model approach, in which each client receives a uniquely personalized model through separate aggregation strategies for their data.
- Utilizing multimodality: It shows if the proposed solution provides multiple data modalities to improve accuracy. This ensures that the model can integrate more than one biometric or behavior feature to authenticate users.
- Using cryptography: It refers to mechanisms that use cryptography, which are integrated with their AI model to improve security and ensure secure aggregation.

**Second: Performance metrics**

The AI-based schemes are compared against several performance metrics. These metrics are chosen because they are the most appropriate metrics that are used in machine/deep learning authentication [100], which are defined as follows:

- Accuracy: It refers to the proportion of correctly identified users or devices in comparison to the total number of authentication attempts.
- False Acceptance Rate (FAR): It refers to the percentage of unauthorized users who are incorrectly granted access by the authentication system. In the biometric field, this metric has a different measure, which is the proportion of the number of false match scores over the total number of impostor match scores. Note that false match scores happen when an impostor’s biometric data (face, fingerprint, etc.) is incorrectly recognized as a legitimate user [15].
- False Rejection Rate (FRR): It refers to the proportion of legitimate users who are incorrectly denied access by the authentication system. In biometric authentication it is the proportion of the number of false non-matching scores over the total number of legitimate match scores. Note that false non-match scores occur when a legitimate user’s biometric (fingerprint, face, etc.) fails to be recognized [15].
- Equal Error Rate (EER): It refers to the point where the FAR and FRR are equal in an authentication system. It represents if the system balances between security (rejecting impostors) and usability (accepting legitimate users).
- True Positive Rate (TPR)/Recall: It refers to the proportion of legitimate users correctly authenticated by the system.
- False Positive Rate (FPR): It refers to the proportion of impostors incorrectly accepted as legitimate users.

Table 8 shows the comparison for AI authentication schemes (behavior/biometric) against the defined criteria. If the scheme satisfies this requirement, then it is marked with (✓); otherwise, it is marked with (×) for not satisfying the requirement.

## 7. Context-Aware Authentication in Federated Learning

**Figure 9:**
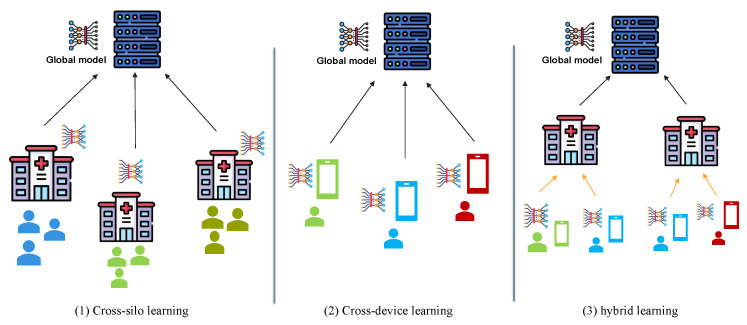
Context-aware FL.

The existing focus for the authentication schemes was based on their underlying technology; however, considering the context in which FL is deployed is an essential dimension. This is important as the authentication process is also affected by the nature of participating clients, system architectures, data distributions, and application domains. In this section, we review these FL authentication schemes through their deployment context, focusing mainly on the following three concepts: cross-silo, cross-device, and hybrid (ad hoc silo) federated learning. Figure 9 shows the differences between each category as follows: (1) cross-silo learning between institutions; (2) cross-device learning across edge devices; and (3) hybrid learning combining device clusters with institutional aggregation. Note that understanding these contextual differences is important for analyzing the practical applicability, limitations, and security features of various authentication approaches. For example, authentication mechanisms that are suitable for resource-constrained, large-scale cross-device FL are completely different from those applied in smaller, trust-based cross-silo environments. Furthermore, hybrid FL introduces a multilayered architecture that requires both device-level and group-level authentication. In the following sections, we review several authentication schemes organized by these FL deployment contexts.

### 7.1. Cross-Silo Learning

It refers to a small number of reliable, trusted institutions, such as hospitals, banks, or companies, that collaborate to train a shared model without sharing their private data. Each silo contains large, diverse datasets with stable, high-capacity computing resources. The main focus of this type is data governance, institutional privacy, and inter-organizational trust. The authentication process of this type usually focuses on ensuring a secure and trustworthy communication between participants, while preventing unauthorized access, tampering, or malicious updates. Due to this trusted nature, cryptography-based and blockchain mechanisms are commonly employed. Ref. [49] provided a continuous authentication scheme that preserves privacy based on user behavior. It involves collaboration between institutional participants, while using a peer-to-peer FL architecture to enhance the authentication process. Several studies fall into this category, for example, [15,50,51], where each study involves the collaboration between organizational or domain-level entities rather than individual devices.

### 7.2. Cross-Device Learning

It refers to a collaborative model training across a large number of edge devices, such as smartphones, smart home devices, and IoT sensors. This setting is less stable than the cross-silo type, as it includes millions of small, resource-constrained connected devices, each of which holds its own local user data. Cross-device learning introduces a different layer of challenges for the authentication schemes, which need to be lightweight and ensure non-IID handling, scalability, and privacy preservation, which can handle frequent client participation and mobility. Authentication schemes in this category usually used biometrics or behavior data, lightweight cryptographic methods, and continuous authentication. Most authentication schemes implement cross-device FL settings. This is because this category is relevant to IoT, UAV, and mobile scenarios. Several mechanisms fall into this category, such as [9,10,12,40,41,43,44,46,47,52,53,54,65,85,93,94,97,101]. This shows that cross-device FL reflects the field’s shift towards user-centric and behavioral authentication, while maintaining privacy and efficiency on constrained devices. Several challenges must be considered when using this approach, like communication bottlenecks, client drop-outs, and vulnerabilities to data and model poisoning.

### 7.3. Hybrid Learning (Ad Hoc Silo)

It refers to systems that combine both cross-device and cross-silo architectures. In this setting, devices are often organized into intermediate clusters or groups, for example, local edge servers, fog nodes, or ad hoc silos, which perform local aggregation before sending the updates to a global server. The authentication process in hybrid learning must operate at multiple levels as follows: (1) ensuring secure device-to-edge interactions, and (2) ensuring edge-to-cloud communications, which may sometimes include peer-to-peer interactions for groups. Authentication schemes in this category are lightweight, and sometimes they provide continuous authentication at the device level with stronger cryptographic or blockchain-based mechanisms at higher aggregation levels.

Several authentication schemes implement this setting. Abdur Rahman et al. [56] proposed a scheme that combines both IoHT edge devices (cross-device) with hospital-level institutional nodes (cross-silo), while blockchain-based decentralized aggregation forms an ad hoc coordination structure. This combination of both learning settings makes the system more realistic and scalable for healthcare environments. Gupta et al. [57] presented another hybrid architecture that balances personalization, privacy, and scalability by distributing training and aggregation responsibilities across different layers. Other schemes that utilize this approach are [13,39,41,42,45,63,74].

This setting represents one of the most promising directions for future FL authentication research, as it integrates devices’ resource constraints with organizational trust, enabling privacy preservation and scalability in heterogeneous environments.

Finally, as presented in this analysis, understanding the system context is important for selecting the most appropriate authentication schemes, ensuring they are aligned with the trust assumptions, resource constraints, and privacy requirements of the federated learning environment. Table 9 shows a comparison of FL authentication schemes, highlighting some key features such as architecture, aggregation algorithm, privacy technique, and domain. In this comparison, privacy techniques mainly concentrate on how the model gradients are protected. These features represent critical factors that influence the design and performance of authentication mechanisms in different FL deployment settings. The choices of these features will directly affect the scalability, trust distribution, and communication behavior within the FL environment.

## 8. Discussion

**Figure 10:**
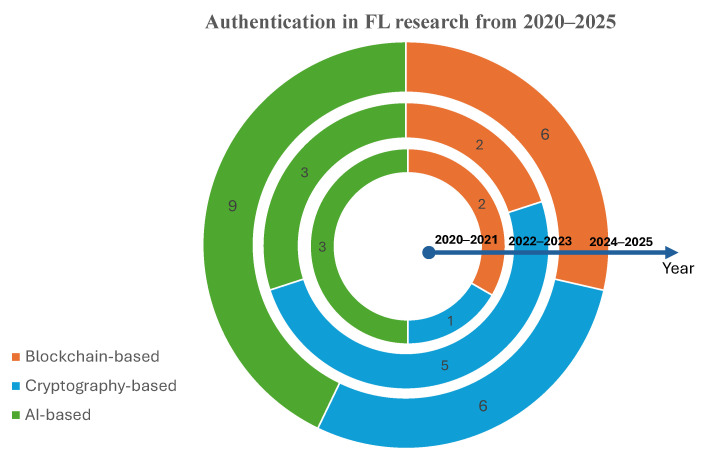
Authentication in FL research development during 2020–2025.

Figure 10 shows the development of authentication in FL in the last 6 years, categorized by their underlying technology. As observed from the figure, the number of research increased during the years, highlighting the growth toward AI-driven personalization and context awareness. There are still some limitations that we observed while conducting our literature review, and they can be outlined as follows:

- The dependency on a single trusted third party for authentication and identification, which is obvious in cryptography-based authentication. Although these mechanisms enhance security, they introduce a single point of failure, scalability concerns, and potential privacy risks. In many cases, the authentication process, anonymity, traceability, and system security depend on this party, assuming that it is totally secure. However, if this entity is breached, misconfigured, or becomes unavailable, the entire authentication framework will be affected. To overcome this issue, zero-trust mechanisms need to be addressed to eliminate reliance on centralized entities and enforce continuous, context-aware authentication at the device level. Additionally, peer-to-peer architectures could be utilized, using behavioral patterns or device properties, without depending on centralized server.
- Cryptographic-based mechanisms are vulnerable to quantum computing, according to Shor’s algorithm [102]. None of the literature solutions address this problem, so it is essential to explore post-quantum cryptographic techniques for authentication, using lattice-based, hash-based, or code-based techniques, etc., which offer resistance against quantum attacks.
- Lack of authentication schemes that integrate both cryptography with behavioral or biometric, which can provide a robust model.
- Multiple research solutions [13,41,44,45] depend on blockchain integration to provide authentication, trustworthiness, and transparency. On the other hand, they do not consider the scalability and efficiency limitations of blockchain.
- The recent literature lacks in providing robust continuous authentication, as only few schemes consider it [9,10,42]. As a result, there is a need to expand research on continuous authentication; especially between devices to enable secure interactions in decentralized IoT environments.
- Most of the existing solutions focus on mitigating poisoning attacks by preventing malicious users from participating in the FL process. However, in many cases, these solutions target mainly unauthorized or fake clients, overlooking scenarios where a registered or authenticated client can intentionally inject poisoned data or manipulate the model. This presents a significant challenge, as insider threats, which are often not addressed. These overlooked vulnerabilities highlight the need for enhanced authentication mechanisms that not only verify user legitimacy but also incorporate continuous monitoring, behavioral analysis, and anomaly detection to detect and prevent adversarial actions, even from insider threats. Future directions must concentrate on developing robust authentication models that fill the gaps and limitations provided in the recent studies.
- Utilizing personalized FL needs to be considered, as there are only few works in this direction [15,43]. The heterogeneity of the biometric data, since it is collected from various environments, sensors, and clients, makes it necessary to consider a personalized model instead of using a single unified model across all clients. This can inspire research to develop scalable and efficient personalized FL frameworks to overcome problems related to biometric recognition.
- Research primarily concentrates on classifying or analyzing schemes based on their underlying technology only, while omitting their overall system context. However, contextual information, such as FL deployment settings, participant roles, and architectural structures, directly influence the trust assumptions, resource constraints, scalability, and security requirements. Accordingly, we provide a complementary classification based on the FL context (cross-device, cross-silo, and hybrid). This will provide a deeper understanding of how authentication mechanisms are shaped and adapted to and their operational setting.
- Several authentication schemes that utilize AI use only accuracy as a metric, which presents a major limitation. Authentication systems require a comprehensive evaluation, as high accuracy alone can be misleading without insights into the FAR, FRR, and EER. Relying on accuracy alone such as in [9,10] can hide critical weaknesses, as a system could have high accuracy but still let in too many impostors (high FAR) or annoy users by rejecting them too often (high FRR). Also the TPR and FPR values are vital for accurately assessing classification performance, especially in real-world deployment scenarios. Authentication systems, especially for continuous user verification, require a comprehensive assessment to capture the potential trade-offs between usability and security. Thus, the table highlights a clear gap in the performance evaluation standards across AI-based authentication research.
- From our observation in Section 7, we found that Federated Averaging (FedAvg) is used in most of the authentication schemes. This preference comes from the FedAvg features, such as simplicity, compatibility with other models, integration with FL tools like TensorFlow Federated and PySyft, and low communication overhead. All these features make FedAvg suitable for devices with limited resources, like IoT, UAV, and edge devices. However, there are many contributions in the federated aggregation field that perform advanced aggregation, for example, FedProx [103], FedNova [104], and other Byzantine resistance or personalized FL methods. This highlights a potential future direction for researchers aiming to develop more robust authentication mechanisms through the integration of advanced and recent aggregation algorithms.

## 9. Datasets and Experimental Environments

Several factors affect the accuracy, robustness, and overall performance of the authentication schemes. The first is choosing the appropriate dataset, which plays a crucial role in evaluating the authentication mechanism because it directly influences the model’s accuracy and behavior. Besides the datasets, the experimental environments where these schemes are implemented also have a major impact on performance. This experimental environments include the number of clients, hardware capabilities, communication settings, and whether the study is simulation-based or implemented on real devices. Understanding both factors is essential for interpreting results objectively and assessing the real-world feasibility of existing authentication schemes. In this section, we introduce and analyze datasets and experimental environments that are used across the reviewed studies.

### 9.1. Datasets Used in the Research

Datasets are one of the fundamental elements in the implementation of authentication schemes in FL. As they determine model performance and reflect real-world distributional challenges, such as non-IID data across clients, selecting the appropriate dataset can be challenging, and the authors must take into consideration several factors, such as the following:

- The relevance to the authentication scheme: Whether the authentication is based on behaviors, biometrics, or cryptographic algorithms.
- The data availability and privacy constraints: Certain types of datasets, such as medical and biometric data, are hard to obtain due to privacy restrictions. In this case, authors usually use simulated or synthetic datasets to implement and test the performance.
- The simulation suitability for FL: The dataset must support partitioning techniques to simulate FL scenarios, like class-imbalance, non-IID distribution, heterogeneous data volume per client, etc.

According to the review and analysis of the literature, we observed the following:

Most of the research used general standard datasets, that are widely available online, like MNIST, CIFAR-10 and CIFAR-100, and Fashion-MNIST. There is also an extended version of MNIST called FEMNIST, which is more suitable for FL algorithms. Although these datasets are not designed specifically for authentication, they are widely adopted due to their availability, simplicity, and support within standard FL simulation frameworks. Furthermore, using these well-known datasets allows researchers to easily compare their results with prior works. They are small, clean, quick to train, and easy to partition across clients. Some of the authentication mechanisms use real datasets, such as in [65], as the authors collected data from a wind turbine in a Chinese farm. Also, [53] collected real physical layer data from Zigbee Zolertia Z1 indoor devices. Zhang et al. [51] use their own RF hardware and backscatter tags to generate the data and test their model.

Several works that depend on behavioral or biometric authentication utilized some publicly available datasets that contain behavioral and biometric data. These datasets are collected from mobile devices or controlled environments, such as touch gestures, keystroke dynamics, motion sensors, face images, and voice recordings. Example of these datasets are UMDAA-02FD [88], UMDAA-01 [105], and MOBIO. These datasets are valuable for continuous and multimodal authentication research. Additionally, in [94], the VoxCeleb dataset is used to evaluate the proposed authentication model. VoxCeleb contains voice recordings of 1251 speakers sourced from YouTube. Moreover, some of the datasets target a specific domain, such as ECG and PPG signals or finger vein images. This type is used in some works [12,15] for physiological and biometric authentication. Other datasets are collected for specific environments, such as [43], which utilized datasets for user interactions in virtual reality environment.

Some datasets are generated through physical experiments and utilized by some research to evaluate their model. For instance, in [40], the authors tested their drone authentication model, based on a radio frequency signal dataset collected from the DeapSECURE training project [106]. This DroneRF dataset was collected using two types of drones (Phantom and Mavic) and contains the following signal features: detection flags, subcarrier spacing, FFT length, CP length, signal power, and symbol time. Another research [48] used a battery state of charge (SOC) dataset collected using controlled physical experiments under different temperatures and loading conditions. This dataset was used to evaluate the performance of SOC estimation models in terms of tracking accuracy, convergence, and robustness. A synthetic dataset is also used for risk-based authentication. For example, in [54], the authors used a synthetic dataset generated by Wiefling et al. [107]. This proposed dataset reflects real-world login behavior based on over 31 million authentication attempts for an online service in Norway. Finally, some authentication research [8,74,108] utilized simulation datasets generated using tools such as SUMO, NS3, and OMNeT++. These simulation tools are often used for IoV environments as they can simulate vehicle’s attributes, such as position, speed, identity, and broadcast messages.

Based on these observation, we found a heavy dependence on standard public datasets that were not originally designed for authentication. This limits the accuracy of evaluating the authentication scheme’s performance in real-world scenarios, as datasets may fail to capture realistic user behavior or security conditions. On the other hand, several works have successfully utilized domain datasets, such as voice (VoxCeleb), face (UMDAA-02FD), or physiological signals (CapnoBase, BIDMC), which provide more realistic data for authentication in FL. Furthermore, we have noticed that there is a need to establish public datasets related to authentication in FL that can handle user diversity, preserve privacy access, and support multimodal signals.

Table 10 provides a description of each dataset, its corresponding domain, and the FL suitability of the publicly available datasets used in the literature. For the FL suitability assessment, we adopt the following criteria:

1. High refers to datasets that provide user separated data, privacy-preservation, and non-IID data.
2. Moderate refers to datasets that have user data availability but limited in heterogeneity or size.
3. Low refers to datasets that have no user distinction, centralized format, or not applicable to FL simulation.

### 9.2. Experimental Environments and Implementation Settings

To better understand the feasibility and scalability of an authentication scheme, it is important to analyze the environments in which it has been implemented. Note that understanding the experimental environment helps in evaluating the practicality of the scheme, as the performance may differ between simulated setups and real hardware. As a result, factors such as device capabilities, communication constraints, dataset size, and number of clients significantly influence the authentication cost and the overall FL performance. One of the most common implementation types is using simulation to provide FL settings, as it is easy and more convenient. However, simulations often overlook critical real-world constraints such as device heterogeneity, limited computational resources, unstable connectivity, and network latency [123]. Implementing in real-devices on platforms such as Raspberry Pi, Jetson Nano, and smartphones often reveals bottlenecks, like CPU limitations, small client numbers, memory usage, communication delays, and client dropout, that do not appear in simulation implementation [124]. Table 11 provides a summary of all the experimental environments that have been used for authentication in FL.

As observed from Table 11, the hardware devices vary widely from powerful GPUs and workstations to resource-constrained devices. This indicates that FL authentication should be able to run across multiple devices. Additionally, some studies tested their model against malicious clients, while others just evaluated their schemes under benign conditions, showing that the level of security evaluation varies between studies. For the implementation type, as we can observed, around 85–90% of the authentication schemes are implemented using simulation, while only few of them are hybrid or real. This indicates that many authentication mechanisms are still evaluated under controlled settings rather than real hardware. Building real-world implementation is very challenging, so we need some solutions to facilitate this implementation type. The following are some suggested solutions:

1. Affordable edge hardware can be used, like Raspberry Pi 4, mobile devices, or NVIDIA Jetson, without the need for a complex setup.
2. Emulators can also be integrated to mimic real hardware and test the scalability, for example, using NS-3, EdgeSim++ for VANETs, or ArduPilot/MINOS for drones.
3. Collaborations can be carried out with industry or academic partners to access real-world test facilities, such as FL pilot environments or innovation labs (e.g., UNDP’s 2025 decentralized AI hackathon or healthcare FL pilots in remote regions like the Amazon rainforest).
4. Containers like Docker, Kubernetes, and Jetson can be used to simplify installation, dependency management and combatibility against different hardware platforms.
5. Finally, building a shared open source testbeds for researchers to remotely access and test their experiment without the need of hardware.

## 10. Open Research Challenges and Future Directions

Federated learning is developing rapidly, and building a secure and trustworthy authentication mechanism that meets FL challenges is not an easy task. Based on the literature there are several contributions in this area that vary in their underlying technology, including AI, blockchain, and cryptography. Each scheme addresses different challenges related to this domain. However, after analyzing and comparing these schemes, we found that there are still some unresolved issues that need to be considered. In this section, we summarize the main open research challenges across this domain, which can serve as a guide for researchers interested in further development.

Post-quantum integration. In Section 5, we observed that all the used cryptography and blockchain mechanisms are based on discrete logarithm problems, such as elliptic curve and Diffie–Hellman or a factorization problem like RSA. None of the research has provided FL authentication using post-quantum cryptography, except [74]. This approach can be a future direction, as more research needs to be integrated in this field. The well-known algorithms (i.e., elliptic curve, RSA, etc.) are vulnerable to attacks by quantum computers, making the exploration of post-quantum cryptography a critical future direction. U.S. National Institute of Standards and Technology (NIST) has selected multiple post-quantum cryptography algorithms for standardization to protect against the potential threats posed by quantum computing [125]. These algorithms are designed to replace current public-key cryptosystems, which are vulnerable to quantum attacks. These algorithms are lattice-based and hash-based. However, these algorithms require computation resources, so there are concerns about how they are going to be implemented in IoT devices. There are several research directions in this area, outlined as follows:

- Developing lightweight post-quantum algorithms that can be used in resource-constrained devices.
- Designing key exchange and management protocols that minimize computational and communication overhead.
- Hardware improvements that can accelerate computations and offload intensive computations from the main processor.
- Using quantum key management, integrated with post-quantum techniques.

Continuous authentication. Federated learning is vulnerable to session hijacking, impersonation, and poisoning attacks, where an adversary could take over an authenticated session and inject malicious updates into the model. Continuous Authentication mitigates these vulnerabilities by verifying the legitimacy of users continuously throughout the session. It will ensure that only authorized and trusted clients participate to the training process. Accordingly, several mechanisms may consider the development of this direction. From Section 5, we observe that there is a gap in this field that needs to be taken into consideration. Several future research can be explored as follows:

- Develop adaptive and intelligent continuous authentication models that can detect Byzantine behavior and poisoned contributions in real time.
- Apply a model that dynamically adjusts authentication levels based on risk assessment.
- The integration of blockchain-based authentication could also enhance the robustness of authentication by decentralizing identity verification and preventing single points of failure.
- Integrating cryptographic techniques with behavioral continuous authentication to build up a secure framework may also be explored.

Device-to-device authentication. The advent of IoT, edge computing, and autonomous systems increased the need for direct communication between devices, and accordingly these communication must be initiated securely. In many FL deployments, devices exchange model updates, control messages, or local decisions without always passing through a central server. In this case, authenticating the user or the client/server link is not sufficient, as the devices themselves must be able to secure and authenticate each other. From our observation of the current literature, behavior/biometric-based authentication is provided only for user authentication [9,10,12,15,43,93,94,101], except the one presented in [40], which authenticates drones by using radio frequency. A future direction would be for researchers to expand this area with more device-to-device authentication, utilizing devices’ biometrics and behaviors. There are several ways to improve this direction as follows:

- Design lightweight protocols between devices, so devices can authenticate each others before sending updates.
- Support dynamic and large-scale groups or clusters, where devices may frequently join, leave, or move (e.g., vehicles, drones, and mobile sensors), requiring group keying, revocation, and fast re-authentication mechanisms.
- Utilize several biometrics or behaviors for devices, like using their hardware imperfections, channel patterns, radio frequency, battery capacity, etc. This will provide an implicit device identity in addition to traditional cryptographic credentials.

Novel aggregation methods. Federated learning depends on aggregation methods; FedAvg is one of the most used algorithms in most of the authentication models. However, it struggles with non-IID data and has security vulnerabilities and high communication cost. Existing aggregation methods often favor clients with larger datasets or more computational resources, leading to biased models. Using an appropriate aggregation method helps authentication models evolve and effectively learn across multiple clients.

There is a need to develop novel aggregation methods that can handle non-IID data diversity and ensure fair training among the clients. Some of the future directions for this field can be the following:

- Weighted aggregation that considers both the data size and quality from each client.
- Incentive mechanisms to fairly distribute training loads and rewards among clients.
- Using decentralized aggregation by transferring the aggregation methods towards the edges or clusters.

Optimization techniques’ integration. To improve the authentication process in FL, several optimization techniques can be integrated to reduce latency and enhance performance, for example, adaptive thresholding via reinforcement learning, secure key management using genetic algorithms, and gradient-based optimization for secure model aggregation. Moreover, game-theoretic models can be leveraged to mitigate poisoning attacks, while multi-objective optimization can balance privacy and authentication accuracy. Several future directions can be considered, such as the following:

- Optimize certificateless key generation using Genetic Algorithms (GAs) or Particle Swarm Optimization (PSO).
- Allocate authentication resources efficiently across edge nodes.
- Model adversarial behaviors and optimally adjust authentication policies to defend against poisoning and impersonation attacks.

Advanced AI integration. Future authentication systems for IoT can utilize advanced AI techniques, such as Reinforcement Learning (RL) and Explainable AI (XAI), to enhance decision-making in dynamic and complex environments. RL can be implemented in authentication models to adjust security levels, instead of using fixed policies based on real-time data, such as network conditions, device types, and user patterns. It can continuously improve the model when integrated with behavioral schemes, as it adapts to evolving patterns over time [126]. On the other hand, XAI can be used to clarify authentication decisions, analyze authentication patterns to detect suspicious activities, and provide transparent security decisions to users [127]. Additionally, Swarm Learning [128] can enhance authentication models by enabling collaborative learning across edge devices while preserving data privacy. It allows peers (nodes) to collaborate directly without a central server. This decentralized approach can be helpful in reducing a single point of failure, dependency, and scalability in authentication.

Zero-trust driven authentication. Zero-Trust (ZT) is a modern security paradigm built on the idea of “always verify, never assume trust”. Systems that are implementing this concept will require continuous checking of identity and authorization for every access request, while considering all users, devices, and network components as untrusted by default and potentially compromised [129]. According to the Cloud Security Alliance (CSA) [130], by the end of 2025, it is expected that 60% of the companies will be or has been shifted to ZT architecture. Authentication is one of the main building blocks for enabling ZT, since every action in this environment must be authenticated, authorized, and continuously validated. Integrating ZT-based authentication into FL provides a robust verification mechanism, as it ensures the client’s identity before uploading their updates and continuously verifies them during the session. Assuming that the parties are untrusted by default helps prevent several attacks, such as poisoning, session hijacking, impersonation, and unauthorized model submissions [131]. Despite the strong alignment between zero-trust principles and the security requirements of FL, the integration of ZT authentication into FL is not well represented in the literature. To the best of our knowledge, only one study proposed by Zhang et al. in [97] is providing an authentication mechanism explicitly designed for FL under a ZT framework. This opens an important future research direction for developing stronger and more adaptive authentication mechanisms in FL.

## 11. Conclusions

Authentication in FL is a recent research topic, and several researchers have proposed solutions in this area. In this survey, we provided an overview of authentication in FL, covering the authentication process, architectural considerations, and security challenges. We reviewed and classified existing authentication methods based on their underlying technologies, including blockchain-based, cryptography-based, and AI-based methods, evaluating their effectiveness and limitations in FL environments. Additionally, we introduced another classification dimension for the literature based on the system context to highlight how authentication schemes adapt to different deployment scenarios. We also discussed the datasets commonly used to evaluate FL authentication mechanisms, emphasizing their characteristics, availability, and limitations. One key observation from our review is the trade-off between decentralization and communication cost. As decentralized designs, especially blockchain, offer strong trust guarantee, but they introduce noticeable communication and synchronization overhead that slows FL rounds. On the other hand, lightweight or partially centralized approaches reduce the communication burden but rely on trusted entities, which makes them less resilient to insider threats. Another takeaway is the balance between authentication strength and scalability. Stronger cryptographic protections improve security but increase latency and limit the number of clients that can participate in each FL round. Meanwhile, scalable schemes simplify the authentication logic or reduce cryptographic complexity, which may weaken their resilience under advanced attacks. Despite the progress in FL authentication, open challenges remain, which include the need for lightweight secure authentication schemes, continuous authentication mechanisms, and adaptive security frameworks. Additionally, there is a need to provide robust schemes that prevent the threats of quantum computing. Future research should focus on developing scalable, privacy-preserving, and attack-resilient authentication models while addressing the dynamic nature of FL.

## Figures and Tables

**Table 1:** Summary of blockchain-based authentication in FL.

Ref.	Year	Main Contributions	Authentication Type	BC Type	Consensus Algorithm	Cryptographic Algorithm Used	Performance Metrics *	Advantages	Limitations
Ji et al. [45]	2023	Lightweight authentication scheme that introduces ZKP and consensus algorithm. Implements an aggregation method based on node contribution and model quality.	Static	Consortium	PBFT	ZKP using Feige Fiat Shamir, Diffie–Hellman, Hash	Accuracy: 93%	+ Provides mutual authentication and traceability + Reduces resilience on central authority + Overcomes problems related to FedAvg by producing a new method	- Does not consider evasion attacks or model inversion attacks - Vulnerable to DoS, impersonation, and session hijacking [58] - Vulnerable to model tampering or node defection [41] - Does not consider non-IID
Feng et al. [14]	2021	Secure authentication framework, using blockchain in FL. It provides cross-domain authentication, using consortium blockchain to record the aggregation model.	Static	Consortium, Private	PBFT, Raft	ECC, Digital signatures (multi and single), Hash, Homomorphic encryption	Accuracy: 94%	+ Decentralized authentication + Cross-domain FL for UAVs + Secure aggregation using homomorphic encryption	- Dependency on KGC in providing private keys - Does not consider malicious user’s behavior [72] - Vulnerable to pooling attacks, collusion attacks, and data tampering [73]
Xiong et al. [63]	2025	Decentralization anonymous authentication, using smart contracts + Shamir’s secret sharing. Uses revocable ring signature for identity and utilizes differential privacy.	Static	Private	PoA	Shamir’s secret sharing [64], Ring signature, hash, ECC, DH	Accuracy: 98.83%	+ Fully decentralized with no trusted key generation center + PoA consensus improves efficiency and filters low-quality models + Anonymous authentication with revocable accountability	- Not resistance to quantum attacks - Accuracy impact from cumulative DP noise with many nodes - High initial overhead for distributed key generation
Chen et al. [13]	2024	Authentication for cross-domain vehicles, that uses blockchain to store authentication records. It uses DBFT to provide fault tolerance and smart contract for secure aggregation.	Static	Private	DBFTs	ECC, Hash	NA	+ Cross-domain authentication + Preserving privacy through anonymity + Reduce communication overhead by 40% + Lightweight	- Single point of failure - Long-term key dependency causes vulnerabilities - Does not provide resilience to insider threats
Liu et al. [41]	2024	Continuous authentication scheme that uses adaptive aggregation algorithm, while implementing chaotic mapping for lightweight key management, and blockchain with smart contracts at fog and cloud servers.	Continuous	Consortium	Raft	Chebyshev chaotic maps, Hash	Accuracy: 98–99%	+ Continuous authentication with trust-aware aggregation + Efficient revocation of malicious nodes + Lightweight protocol using Chebyshev maps	- Suffer from insider model poisoning - Requires trusted fog nodes
Abdur Rahman et al. [56]	2020	Decentralized mechanism for FL, using blockchain and homomorphic encryption to ensure data provenance and security.	Static	Private	PoA, RAFT	Digital signature, Hash, AES, RSA for homomorphic encryption, Paillier algorithm	Accuracy: 91.77%	+ Strong end-to-end encryption + Secure transfer learning + Smart contract provenance tracking + Trust and aggregation management using blockchain	- No clear clarification for threat model used [19] - Heavy computational resource for edge nodes [69] - TEE requires trusted hardware
Gupta et al. [57]	2024	Distributed authentication mechanism for personalized recommendation systems, utilizing homomorphic encryption to preserve privacy.	Static multi-factor	Not mentioned	Not mentioned	Homomorphic encryption Hash	Root Mean Squared Error: 0.95–1.15 Fit time: 0.2–19 s	+ Blockchain ensures auditability and trust + Hierarchical design provides scalability + Handles collaborative filtering use case	- Significant delays caused by linear search [71] - Less suitable for high-demand environments [71] - No security analysis - No protection against inference attacks
Ahmed and Anisi [74]	2025	Post-quantum authentication scheme for FL, that utilizes XMSS/LMS and PUF security to authenticate vehicles. It also uses blockchain for storing credentials and off-chain for real-time authentication.	Static	Consortium	PBFT	XMSS, LMS, AES-GCM, Hash	Throughput: 142.6 kbps End-to-end delay: 7.2 ms Packet Delivery Ratio (PDR): 94.3%	+ Quantum resistance + Prevent insider poisoning by anomaly detection + Hardware authentication +Reduce latency using off-chain	- No backward secrecy for the renewal key - Edge and charging stations devices are not considered - Edge node can poison results - Using accuracy metrics only maybe insufficient
Fan et al. [44]	2023	Decentralized lightweight anonymous authentication framework DAFL, using blockchain in FL. Provides decentralization by using DAG structure.	Static	Consortium (DAG-structure)	Committee validation	ECC, Dynamic Accumulators, Lightweight Digital Signatures, Hash	Accuracy: 82.5%	+ Decentralized and lightweight + Batch verification reduces computation overhead + Supports anonymity and traceability + Improves global model accuracy in FL	- Lacks strong accountability for malicious clients [77] - Local model parameters transmitted in plaintext [75] - Limited privacy protection (medium) [76] - No data minimization - No monitoring for real-time detection of malicious activity [75] - Does not provide perfect forward secrecy [58]

* The performance metrics presented should not be interpreted as direct comparisons between approaches, but rather as performance values achieved within each study’s original experimental setup and constraints. For additional details about the testing environments, please refer to Section 9.2.

**Table 2:** Computational complexity of blockchain-based authentication schemes in FL.

Scheme	Client	Edge Node	Server Node
Ji et al. ^*a*^ [45]	$1T_{\mathrm{FFS}}+1T_{\mathrm{DH}}+1T_{\mathrm{AES}}$	$1T_{\mathrm{FFS}}+1T_{\mathrm{DH}}+1T_{\mathrm{AES}}$	Not mentioned
Feng et al. ^*a*^ [14]	$1T_{\mathrm{DSA}}+1T_{\mathrm{hash}}+1T_{\mathrm{HE}}$	$2T_{\mathrm{BP}}+1T_{\mathrm{BC}}+1T_{\mathrm{HE}}$	Not mentioned
Xiong et al. [63]	$nT_{\mathrm{BP}}+T_{m}+T_{\mathrm{hash}}$	$nT_{\mathrm{BP}}+nT_{m}+T_{\mathrm{hash}}$	Not mentioned
Chen et al. ^*b*^ [13]	$18T_{\mathrm{hash}}$	$1T_{\mathrm{ECDSA}}+20T_{\mathrm{hash}}+1T_{\mathrm{BC}}$	$1T_{\mathrm{BC}}+1T_{\mathrm{ECDSA}}+9T_{\mathrm{hash}}$
Liu et al. [41]	$4T_{\mathrm{cm}}+6T_{\mathrm{hash}}$	$4T_{\mathrm{cm}}+5T_{\mathrm{hash}}$	$2T_{\mathrm{cm}}+12T_{\mathrm{hash}}+1T_{\mathrm{BC}}$
Abdur Rahman et al. ^*c*^ [56]	$1T_{\mathrm{HE}}+1T_{\mathrm{AES}}$	Not applicable	$1T_{\mathrm{HE}}+1T_{\mathrm{BC}}+1T_{\mathrm{AES}}$
Gupta et al. ^*d*^ [57]	$1T_{\mathrm{HE}}+1T_{\mathrm{hash}}$	$3T_{\mathrm{hash}}$	$1T_{\mathrm{HE}}+1T_{\mathrm{BC}}$
Ahmed and Anisi ^*d*^ [74]	$2T_{\mathrm{HMAC}}+3T_{\mathrm{hash}}+2T_{\mathrm{XMSS}}+1T_{\mathrm{AES}}+1T_{\mathrm{PUF}}$	$2T_{\mathrm{HMAC}}+2T_{\mathrm{hash}}+3T_{\mathrm{XMSS}}+1T_{\mathrm{AES}}+1T_{\mathrm{PUF}}$	Only for cross-domain ZKP
Fan et al. ^*d*^ [44]	$3T_{m}+4T_{\mathrm{hash}}$	Not applicable	$3T_{m}+2T_{\mathrm{hash}}+1T_{a}$

^*a*^ The authentication process is tightly dependent on the blockchain trust framework. ^*b*^ This cost is a sum of both initial and handover authentication. ^*c*^ This scheme used TEE for blockchain, smart contracts, and aggregation. ^*d*^ This includes both registration and authentication.

**Table 3:** Symbols and their corresponding cryptographic operations.

Symbol	Operation
$T_{\mathrm{AES}}$	AES encryption
$T_{\mathrm{Sig}},\;T_{\mathrm{DSA}},\;T_{\mathrm{ECDSA}}$	Digital signature
$T_{m}$	Point multiplication (ECC)
$T_{\mathrm{PUF}}$	Physical unclonable function
$T_{\mathrm{cm}}$	Chaotic map operation
$T_{\mathrm{BC}}$	Blockchain write
$T_{\mathrm{BP}}$	Bilinear pairing
$T_{\mathrm{HE}}$	Homomorphic encryption
$T_{\mathrm{XMSS}}$	XMSS post-quantum signature
$T_{a}$	Point addition (ECC)
$T_{\mathrm{HMAC}}$	HMAC operation
$T_{\mathrm{FFS}}$	Feige–Fiat–Shamir (FFS) zero-knowledge proof

**Table 4:** Comparing blockchain-based authentication.

Ref.	[45]	[14]	[63]	[13]	[44]	[41]	[56]	[57]	[74]
Security requirements									
Mutual authentication	✓	×	✓	✓	✓	✓	×	×	✓
Continuous authentication	×	×	×	×	×	✓	×	×	×
Anonymity	×	×	✓	✓	✓	✓	×	✓	×
Traceability	✓	✓	✓	×	✓	✓	✓	✓	✓
Forward secrecy	✓	✓	✓	×	NA	✓	NA	×	✓
Backward secrecy	✓	✓	✓	×	NA	✓	NA	×	×
Decentralization	✓	✓	✓	×	×	×	✓	✓	✓
Secure aggregation	✓	✓	✓	×	×	×	✓	✓	×
Security attacks									
MITM prevention	✓	✓	✓	✓	✓	✓	✓	×	✓
Poisoning prevention	✓	✓	✓	×	×	×	×	×	✓
Impersonation prevention	×	✓	✓	✓	✓	✓	×	×	✓
Quantum attacks prevention	×	×	×	×	×	×	×	×	✓

NA: not applicable.

**Table 5:** Summary of cryptography-based authentication in FL.

Ref.	Year	Main Contributions	Type of Authentication	Multi-Factor Authentication	Trust Anchor	Cryptographic Algorithm Used	Advantages	Limitations
Deebak & Hwang [39]	2023	Lightweight authentication L2FAK, provides two-factor authentication (possession- and knowledge-based) and adds additional security using devices’ attributes.	Static	Yes, two-factor	Authentic Gateway	ECC, Hash	+ Secure against unauthorized modifications, session stealing, insider threats, and DoS + Utilizes FL to apply an extra layer of authentication + Two-factor authentication + Efficient data transmission	- Lacks in addressing some IoMT attacks, like spoofing, alteration, and traffic attacks [79] - Depends on static attributes, rather than behaviors - It is proposed for IoMT, but the dataset used is not related to medical
Feng et al. [42]	2024	Continuous authentication for VANET, by employing non-interactive zero-knowledge proof to validate clients and provides a distributed architecture.	Continuous	No	TA	ECDH, ZKP, Short Group Signatures	+ Reduced communication/computation overhead + Preserves privacy by using FL aggregation and zero-knowledge + Continuously checks legitimacy during the session	- Fails to address both revocability and anonymity [80] - Single point of failure and TA bottleneck - Vulnerable to poisoning attacks
Li et al. [46]	2023	Certificateless authentication for 6G communications, using hybrid key mechanism to enhanced security.	Static	No	TRA, KGC	ECC, Certificateless, Hash, Digital signatures	+ Suitable for resource-constrained devices + Eliminates certificate management overhead + Ensures integrity and trustworthiness	- Single point of failure - Does not address inference attacks, model inversion or side-channel attacks - Provides only theoretical proof
Huang et al. [47]	2024	Provides certificateless authentication protocol for IIoT. Utilizes batch verification using group key. Uses TESLA (Timed Efficient Stream Loss-tolerant Authentication Protocol) to distribute global model parameters securely.	Static	No	TA	ECC, Certificateless, CTR	+ Ensures non-repudiation + Prevent attackers from linking multiple data submissions + Group-based authentication + Efficient authentication	- Vulnerable to insider users poisoning - Single point of failure - No confidentiality for the transferred parameters
Yuan et al. [48]	2023	Authentication protocol to preserve privacy in VANET. It implements unlinkable pseudonyms to anonymize vehicles. CRT is used to help in defining and managing communication groups among vehicles.	Static	No	TRA	ECC, Certificateless Signature, CTR	+ Unlinkable pseudonyms ensure vehicle anonymity in federated learning + Simplifies key management by removing certificate dependency + CRT enables efficient dynamic group key updates	- Public key replacement flaw enables malicious signature creation - TA fails to identify malicious vehicles due to pseudonym randomness [82] - Group updates become computationally expensive with frequent vehicle changes [82]
Liu et al. [55]	2024	Certificateless authentication scheme for IoMT, using blind masking and re-encryption techniques	Static	No	KGC, TRA	Certificateless Signature, Hash	+ Prevents gradient leakage + Provides fault tolerance + Secures IDs with pseudonyms	- Vulnerable to quantum attacks - Non-IID problem is not handled - Still vulnerable for poisoning attack
Wang et al. [50]	2023	Authentication mechanism between Charging Stations and Charging Station Provider, ensuring integrity of local updates used for energy demand prediction.	Static	No	KGC	Identity-based signatures with bilinear pairings, Hash, AES	+ Supports batch authentication + Trust/reward system + Defends FL attacks	- Does not provide anonymity - Single point of failure [44] - Vulnerable to DoS and impersonation attacks [58]

TA: Trusted Authority; TRA: Tracing Authority; KGC: Key Generation Center.

**Table 6:** Comparing cryptography-based authentication.

Ref.	[39]	[42]	[46]	[47]	[48]	[55]	[50]
Security requirements							
Mutual authentication	✓	✓	✓	✓	✓	×	✓
Continuous authentication	2FA	✓	×	×	×	×	×
Anonymity	✓	✓	✓	✓	✓	✓	×
Traceability	×	×	✓	✓	✓	✓	✓
Forward secrecy	✓	✓	✓	✓	×	✓	✓
Backward secrecy	×	✓	✓	✓	×	✓	✓
Decentralization	✓	×	×	×	×	×	×
Secure aggregation	×	✓	×	×	×	✓	×
Security attacks							
MITM prevention	✓	✓	✓	✓	×	✓	✓
Poisoning prevention	✓	×	✓	✓	✓	×	✓
Impersonation prevention	×	✓	✓	✓	✓	✓	✓
Quantum attacks prevention	×	×	×	×	×	×	×

2FA: Two-factor authentication.

**Table 7:** Summary of AI-based authentication in FL.

Ref.	Year	Main Contributions	Type of Authentication	ML/DL Algorithm	Performance Metrics *	Advantages	Limitations
Wazzeh et al. [9]	2024	Provides a continuous authentication for mobile crowdsourcing. It integrates transfer learning + warmup techniques. Using behavioral biometrics data.	Continuous/user-to-device Behavior	CNN	Accuracy: 80%	+ Handle non-IID + Using Transfer learning and a warmup phase improves accuracy + Scalability + Continuously verify users during the session	- FedAvg aggregates a single global model for all users, cannot handle diversity - High-dimensionality data like images have slow convergence - Vulnerable to model/user poisoning or adversarial attacks
Cheng et al. [43]	2024	First FL-based authentication framework for VR. Selects the best biometric modality for users to optimize authentication.	Static/user-to-device Behavior	CNN	Accuracy: 98% EER: 1.3% TPR@0.5% FPR: 80% TPR@ 0.1% FPR: 72%	+ Multimodal Optimization + Low Latency + Mitigate mimicry, impersonation, and model intervention + Choosing appropriate behavior for each user (Personalized)	- Vulnerable to replay/synthesis attacks - Leaks user’s behavior information
Yazdine-jad et al. [40]	2021	Provides an authentication for drones in FL, using DNN, with radio frequency.	Static/device-to-device Biometric	DNN	Accuracy: 94.16% Precision: 95.8% F1-measure: 93.7% Recall: 92.11%	+ Provides devices’ authentication + Privacy + Secure aggregation using homomorphic encryption	- Vulnerable to poison, Byzantine attacks and session hijacking - Does not consider RF spoofing and signal interference - High computation of homomorphic encryption for drones
Lu et al. [65]	2022	Provides authentication using facial recognition for operators of wind turbines. The main contribution is managing the class imbalance in this field.	Static/user-to-device Biometric	CNN	Accuracy: 91.77% AUC: 92%	+ Handles class imbalance via self-monitor loss + Prevents gradient leakage + Real-world implementation on wind turbines + Low overhead	- Has problems with diverse label subspace - Suffers from spoofing and inside poisoning attacks - Requires auxiliary data or estimation for reweighting
Wazzeh et al. [10]	2024	Provides a continuous authentication CRSFL. Addresses client’s selection during split FL, while clustering clients. Optimize resource allocation using Genetic Algorithm.	Continuous/user-to-device Behavior	CNN (VGG16, FacialImageNet, Net4L)	Accuracy: 89%	+ Efficient client selection for training + Reduces client dropout by selecting only capable devices + Parallel split learning improves speed over traditional FL + Handle devices heterogeneity	- Does not provide secure aggregation, which may leak sensitive data - GA client selection increases computational complexity - Multi-objective optimization problem is NP-hard, increasing complexity as the number of clients increases
Lian et al. [15]	2023	Provides an authentication scheme based on Finger vein (FV). Introduces a novel aggregation algorithm (FedWPR). Used personalized model for aggregation.	Static/user-to-device Biometric	CNN (MobileNetV2)	Accuracy: 99.85% EER: 0.07% TAR@FAR = 0.01: 99.65%	+ Addresses non-IID data and client imbalance contribution + Uses FedWPR aggregate model + First personalized FL framework (model for each user) + Preserves privacy	- The model can cause significant waiting times for clients [91] - Does not utilize the idle period [91] - Maintaining N personalized models (one for each client) on the server can be computationally expensive - No optimal solution [92]
Coelhoa et al. [12]	2023	Provides an authentication mechanism for users in FL, using both (ECG) and(PPG) signals.	Static/user-to-device Biometric	CNN	Accuracy: 99.27% FAR: 0.12% FRR: 64.68% Precision: 73.20% Recall: 35.31%	+ Using multimodal biometric improves robustness, and prevents spoofing + ECG, PPG are unique, difficult to replicate, and inherently linked to the individual + No direct user intervention	- Training complex CNNs locally could be resource-intensive [51] - Vulnerable to session attacks - FedAvg not optimal for non-IID data leading to suboptimal model convergence or bias global model [16] - Biometric data are sensitive and cannot be reset if compromised [99]
Wehbi et al. [85]	2024	Provides a mutual authentication based on a trust score mechanism. Clients’ trust scores depend on their behavioral resource usage patterns.	Continuous/device-to-device Behavior(trust-based)	DNN	Accuracy: 88.69% AUC: 95% TPR: 93.75% FPR: 4.17%	+ Mutual trust-based authentication for both clients and servers + Prevents session hijacking + Prevents malicious server participation + Reduces untrustworthy client selection	- Vulnerable to collusion and impersonation attacks - Cold-start problem for new participants - Potential overhead in large-scale deployments
Zhang et al. [97]	2025	Provides authentication based on spectrogram. Based on novel CNN with anchor loss function.	Continuous/device-to-device Biometric	CNN	Accuracy: 80% AUROC: 0.7	+ Zero-trust framework + Can classify known and unknown UAVs + Considers the security in all layers (data, network, and task) + Perform well in noisy environments	- Same manufacturer drones is misclassified - Non-IID is not considered - Vulnerable to poisoning attacks
Istiaque Ahmed et al. [53]	2024	Provides authentication based on devices’ physical layer features, such as: link quality, temperature, RSSI, MAC address, etc. Using trust-aware to assess devices’ reliability.	Static/device-to-device devices’ attributes	ANN	Accuracy: 99.79% TPR: 99.79% Precision: 99.75% Recall: 99.93% FAR: 0.07, FRR: 0.21 F1-score: 99.77%	+ Real Zigbee IoT dataset + Device-to-device authentication + Dynamic trust evaluation based on physical layer attributes	- No noise handling - Vulnerable to interference, temperature drift and multi-path effects - No secure aggregation
Oza and Patel [93]	2021	Provides Federated Active Authentication (FAA) to continuous user authentication across mobile devices using face recognition	Continuous/user-to-device Biometric	SVM, CNN (VGG16)	Accuracy: 99.8%	+ Low communication overhead + Using multi-class classification + Novel Gaussian sampling for solving non-IID	- Server classifier can still be exposed - It still shares some user features with the server [52] - Poor generalization for users with limited data [10] - No evaluation for mobile active attacks, like spoofing and Sybil attacks [53]
Hosseini et al. [94]	2020	Proposes FedUA, a privacy-preserving framework using FL with random binary embeddings for user authentication across smart devices.	Static/user-to-device Biometric (voice)	CNN	TPR: 80% FPR: 0.16%	+ Using random binary vectors to protect user’s real embeddings or feature statistics + High scalability + Implements a warmup phase	- Vulnerable to inversion attacks and model poisoning - Static threshold tuning per user - Not sufficient performance evaluation
Fereidouni et al. [54]	2024	Proposes F-RBA, a FL framework using deep autoencoders for risk-based behavioral authentication, enabling privacy-preserving and adaptive detection of anomalous login behavior across non-IID user data.	Continuous (context- and behavior-based risk evaluation)	Deep Autoencoder (AE)	Accuracy: 92.4% Recall: 88.3% Precision: 94.7% F1-score: 90.9%	+ Real-time continuous assessment + Cold-start friendly + IPFS for decentralized history + Cross-device profiling	- Suffers from fairness issues and bias global model [86] - Vulnerable to model poising attack [86] - May face issues in performance and privacy within public clouds
Zhang et al. [51]	2024	Provides a lightweight authentication by utilizing backscatter tags to reflect signals and generate a unique signature for each device.	Static authentication (Device biometric using backscatter identity)	DNN RF, DT, SVM, NB, LR	Accuracy: 98.4% FPR: 0.59% TPR: 97% AUCROC: 93.78%	+ Lightweight suitable for limited resources devices + Uses passive backscatter tags to create unique device identity + Novel aggregation method resists poisoning by weighting tag count	- One-way authentication - No handover or roaming support across domains [98] - Vulnerable to eavesdropping and model inversion - Requires special backscatter tags for each device

* The performance metrics presented should not be interpreted as direct comparisons between approaches, but rather as performance values achieved within each study’s original experimental setup and constraints. For additional details about the testing environments, please refer to Section 9.2.

**Table 8:** Comparing AI-based authentication.

Ref.	[9]	[43]	[40]	[65]	[10]	[15]	[12]	[85]	[97]	[53]	[93]	[94]	[54]	[51]
Security requirements														
Continuous authentication	✓	×	×	×	✓	×	×	✓	×	×	✓	×	✓	×
Non-IID Handling	✓	✓	×	×	✓	✓	×	✓	×	✓	✓	✓	✓	×
Personalized model	×	✓	×	×	×	✓	×	×	×	×	×	×	✓	×
Multimodality	✓	✓	×	×	×	×	✓	✓	×	✓	×	×	×	×
Cryptography	×	×	✓	×	×	×	×	×	✓	×	×	×	×	×
Performance metrics														
Accuracy	✓	✓	✓	✓	✓	✓	✓	✓	✓	✓	✓	-	✓	✓
FAR	-	-	-	-	-	✓	✓	-	-	✓	-	-	-	-
FRR	-	-	-	-	-	✓	✓	-	-	✓	-	-	-	-
EER	-	✓	-	-	-	✓	-	-	-	-	-	-	-	-
TPR	-	✓	✓	-	-	-	✓	✓	-	✓	-	✓	✓	✓
FPR	-	✓	-	-	-	-	-	✓	-	-	-	✓	-	✓

**Table 9:** Comparing authentication schemes in FL based on context-aware factors.

Ref.	FL Context	Architecture	Aggregation Algorithm	Privacy Technique	Domain	System Context (Influencing Factors)
Monschein et al. [49]	Cross-silo	Peer-to-peer (P2P) fully distributed	P2P aggregation	Multi-Party Computation(MPC)	Cryptocurrency exchange platforms (CEPs)	Data governance requirements and multi-level organizations
Wang et al. [50]	Cross-silo	Centralized	FedAvg	-	Electric vehicle infrastructures	Dynamic and variable energy demand and heterogeneous environment
Zhang et al. [51]	Cross-silo	Hierarchical partially distributed	FedAvg	-	IoT	Wireless channel variability and environment interference
Lian et al. [15]	Cross-silo	Centralized	FedWPR	-	Biometric devices (finger vein)	Non-IID and unbalanced data across institutions, heterogeneous devices and imaging setups, data-island problem
Coelho et al. [12]	Cross-device	Centralized	FedAvg	-	IoHT	Limited resources, biometric heterogeneity and non-IID, sensitive data
Wazzeh et al. [52]	Cross-device	Centralized	FedAvg + warmup	-	IoT	Clients’ data variability and label uniqueness
Istiaque Ahmed et al. [53]	Cross-device	Hierarchical partially distributed	FedAvg with trusted weight aggreagation	-	IoT	Resource constraints, dynamic, and unreliable IoT devices
Zhang et al. [97]	Cross-device	Centralized with zero-trust	FedAvg under model verification	-	6G UAVs	Dynamic and mobile UAV clients with noisy RF environment, need for lightweight models, and low-latency inference
Fereidouni et al. [54]	Cross-device	Centralized	FedProx	-		Heterogeneous and non-IID behavioral data across distributed
Liu et al. [55]	Cross-device	Centralized	FedAvg	Blind Masking using Certificateless Proxy Re-Encryption (CL-PRE)	IoMT	Resource constraints, connection unstable, and data-sensitivity in IoMT environment
Oza and Patel [93]	Cross-device	Centralized (Split Learning influence)	FedAvg and split learning	-	Mobile application	Non-IID, sensitive, and private biometric data + real-time constraints in users’ mobiles
Cheng et al. [43]	Cross-device	Centralized	FedAvg	-	Virtual Reality	Mobility, multi-motion data, highly non-IID and need for low latency
Hosseini et al. [94]	Cross-device	Centralized	FedAvg	-	Smart devices	Resource constraints and heterogeneous and sensitive user data from smart devices environment
Lu et al. [65]	Cross-device	Centralized	FedAvg with partial parameter selection	Gradient masking via Gaussian noise	Wind turbine	Class-imbalance data and privacy concerns
Wehbi et al. [85]	Cross-device	Hierarchical	FedAvg	-	Smart cities	Heterogeneous smart cities devices, untrusted servers/clients
Yuan et al. [48]	Cross-device	Centralized	FedAvg	-	VANET	Heterogeneous, mobility, untrusted VANET environment
Fan et al. [44]	Cross-device	Distributed blockchain (DAG)	Not specified	-	IoT	Untrusted, dynamic environments
Huang et al. [47]	Cross-device	Centralized	FedAvg	-	IIoT	Devices constraints, heterogeneity, and dynamic
Li et al. [46]	Cross-device	Centralized	FedAvg	-	6G	High mobility, large number of IoT, low latency, decentralized trust, and resource constraints
Wazzeh et al. [9]	Cross-device	Centralized	Weighted FedAvg with warmup + transfer learning	-	Mobile crowdsourcing	Non-IID behavioral data, privacy sensitivity heterogeneous devices, and communication cost
Wazzeh et al. [10]	Cross-device	Hierarchal	FedAvg + parallel split learning within clusters	-	Mobile/IoT	Device heterogeneity and limited resources, non-IID behavioral data, and low communication overhead
Sakka et al. [101]	Cross-device	Distributed blockchain	FedAvg	ZNP and AES	Healthcare (ADHD monitoring)	Shared device environment, GDPR compliance, decentralized identity verification, limited resource devices, and trust requirements
Yazdinejad et al. [40]	Cross-device	Centralized	FedAvg	Homomorphic encryption	Drones	Dynamic drone mobility, heterogeneous RF data, scalability, privacy preservation, and wireless communication constraints
Abdur Rahman et al. [56]	Hybrid	Distributed blockchain	FedAvg (secure gradient aggregation)	Full Homomorphic Encryption Differential Privacy (DP) SGX Trusted Execution Environment (TEE)	IoHT	Healthcare regulations and heterogeneous, private, provenance environment
Gupta et al. [57]	Hybrid	Distributed blockchain	Distributed FedAvg	Homomorphic Encryption	Consumer electronics	Heterogeneous, private sensitive consumer data
Liu et al. [41]	Hybrid	Distributed blockchain	Adaptive aggregation model	-	VANET	High mobility and dynamic topology in VANET
Xiong et al. [63]	Hybrid	Distributed blockchain	FedAvg	Differential privacy	IoT	Untrusted heterogeneous IoT/edge network
Ahmed and Anisi [74]	Hybrid	Distributed blockchain	Secure aggregation using Shamir’s Secret Sharing	Differential privacy+ ZKP	V2G/IoV	Heterogeneous, mobility, and untrusted V2G domain
Chen et al. [13]	Hybrid	Distributed blockchain	FedAvg via smart contract	Symmetric encryption using session key	Consumer electronics- autonomous driving	Mobility, cross-domain vehicle handover, hierarchical coordination, and lightweight computation
Ji et al. [45]	Hybrid	Distributed blockchain	Adaptive FedAvg with weights	Differential privacy and ZKP	IoT	Resource constraints, data heterogeneity, node reputation, and trust
Deebak & Hwang [39]	Hybrid	Hierarchical	FedAvg	AES	IoT-healthcare	Device constraints, heterogeneity, and sensitive medical data
Feng et al. [42]	Hybrid	Hierarchical	FedAvg	Masking and ZKP	VANET	High mobility, dynamic, edge coordination with semi-honest/malicious models

**Table 10:** Datasets used for authentication in FL.

Dataset Name	Publisher	Domain	FL Suitability	Description	Used In
MNIST	Kaggle	Handwritten digits	Low	It is a large database containing around 60,000 for training hand written digits and around 10,000 for testing	[9,14,39,41,42,44,45,50,52,59,63,85,109]
Federated Extended MNIST (FEMNIST)	LEAF benchmark	Handwritten digits/image classification	High	Partitioned by user ID for around 3500 users, and it is divided into 62 different classes	[9,14,52]
Fashion-MNIST	Kaggle	Fashion Object classification (Grayscale images)	Low	It contains 70,000 grayscale images of cloth items classified into 10 different classes, which are collected from Zalando’s fashion products	[39,50,61,85]
CIFAR- 10 and CIFAR-100	University of Toronto	Object classification (images)	Low	CIFAR-10 consists of 10 different categories that have in total 60,000 colored images. The CIFAR-100 is the same as 10 but it is classified into 100 different classes	[9,41,42,44,45,50,52,59,63]
UMDAA-02FD	University of Maryland	Face detection	High	It contains face images captured from front-facing smartphone cameras of 48 users in real case scenarios	[9,10,93]
MOBIO	Idiap research institution	Face/voice biometric data	High	It contains data (voice + images) collected from 150 users using mobile devices and laptops	[93]
CapnoBase	IEEE TBME Respiratory Rate	Physiological signals (ECG/PPG)	Moderate	It contains 42 different signals including: photoplethysmogram/pulse oximetry (PPG), capnography and electrocardiogram (ECG) from different patients	[12]
BIDMC	PhysioNet	Physiological signals (ECG/PPG)	Moderate	It contains 53 record from different ICU patients and is manually annotated for ECG and PPG signal recordings	[12]
TROIKA	Zhang et al. [110]	Physiological signals (ECG/PPG)	Moderate	It contains PPG, accelerometer, and reference ECG signals from 12 subjects during exercise	[12]
VoxCeleb	University of Oxford	Speaker recognition/voice biometrics	High	It contains audio recordings for more than 1200 speakers; these data are collected from YouTube	[94]
RBA Synthetic Dataset	Wiefling et al. [107]	Behavioral login authentication	Moderate	It is a synthetic dataset reflecting 31 M login attempts from millions of users based on real-world statistics	[54]
6 virtual reality datasets (Throw, Point, Walk, Watch, Type, and Grab)	Throw [111] Point, Type, Grab, Walk [112] Watch [113]	Users behaviors in VR	High	Six publicly available datasets for evaluating multimodal behavioral biometrics in VR (e.g., hand/head/gaze movements)	[43]
SOC battery dataset	University of Maryland	Battery data	Moderate	It is collected from real physical experiments with two types of OCV tests (low-current and incremental-current) at 3 temperatures	[48]
Zigbee Physical-Layer Dataset	Ahmed et al. [114]	IoT/RF authentication, using radio parameters (RSSI, LQI, temperature, etc.)	High	It contains physical-layer attributes for Zigbee devices, which are collected in a real-world indoor environment	[53]
DroneRF dataset	DeapSECURE project	Radio frequency (RF) data for drones	High	This dataset contains RF signal parameters, such as CP length, subcarrier spacing, etc. These data are collected from two types of drones, called Phantom and Mavic	[40]
9 public datasets for finger vein, namely, HKPU-FV, SDUMLA-HMT, THU-FVFDT, FV-USM, PLUSVein-FV, MMCB-NU-6000, SCUT-FV, UTFVP, and VERA	MMCB-NU-6000 [115] THU-FVFDT [116] FV-USM [117] SDUMLA-HMT [118] VERA [119] SCUT-FV [120] PLUSVein-FV [121] UTFVP [122] HKPU-FV [115]	Finger vein biometrics	High	These nine datasets are either specific for finger vein biometric data only, like THU-FVFDT, VERA, etc., or they can combine multiple biometrics, including finger vein, such as SDUMLA-HMT. These data are collected in real-time environments from users through near-infrared imaging devices that can capture the vein features under varying lighting or pressure conditions	[15]

**Table 11:** Experimental environments used for authentication in FL.

Ref.	Implementation Type	Hardware/Platform	FL Tool	Number of Clients	Malicious Clients
Wang et al. [50]	Simulation	-	Google TensorFlow	-	25% malicious
Zhang et al. [51]	Real-device implementation (prototype)	Backscatter tags (AGLN250 FPGA + ADG902 switch), server (PC), Edge server implemented on USRP + computer	Custom FL	4 IoT clusters (each cluster = several IoTDs	Different semi-honest + Powerful attackers
Lian et al. [15]	Simulation	-	-	9 clients	-
Coelho et al. [12]	Simulation	Pulse oximeter, Finger oximeter, Wet ECG chest	TensorFlow, TensorFlow Federated	20 nodes (clients)	-
Wazzeh et al. [52]	Simulation	GPU (GTX 1660 Super), 16 CPUs at 2.90 GHz	FedML Github	-	-
Istiaque Ahmed et al. [53]	Hybrid	Zigbee Zolertia Z1 nodes, 802.15.4 radios	Custom FL	-	Spoofing, Sybil
Zhang et al. [97]	Hybrid	USRP B210, RF signals from various UAVs, Workstation Ubuntu (22.04) and AMD Ryzen 9 7900, 3D CPU, 64 GB RAM, and an NVIDIA GeForce RTX 4070 Ti GPU	Python + PyTorch	5 clients	-
Fereidouni et al. [54]	Simulation	Intel(R) Core(TM) i9-9880H, 16 GB RAM, 1 TB SSD, macOS (Conda)	TensorFlow Federated	500 users	-
Liu et al. [55]	Simulation	Intel Xeon CPU E3-1230 V2 @ 3.30 GHz, 16 GB RAM	Custom FL	-	-
Oza and Patel [93]	Simulation	Intel Core i9, 16 GB RAM, VGG16 (feature extractor)	Custom FL	25 enrolled users	Unauthorized/unknown users (variable)
Cheng et al. [43]	Simulation	RTX 3080 GPU, Nvidia Jetson Xavier NX	PyTorch	41 users	Impersonation, Mimicry attacks
Hosseini et al. [94]	Simulation	-	Custom FL	128, 256 and 512	-
Lu et al. [65]	Hybrid	Wind turbine farms (China), NVIDIA GeForce GT 720, Intel Core i3 (Two-core 1.8 GHz)	PyTorch	-	Malicious access
Wehbi et al. [85]	Simulation	Different IoT devices (RAM 100–1300 MB, CPU 50–1000 MIPS, Bandwidth 50–1300 Mbps)	ModularFed	200 clients	Poisoning attacks, Untrustworthy clients
Yuan et al. [48]	Simulation	Intel(R) Core(TM) i5-12500H, 16 GB RAM, RTX 3050 GPU, Windows 11	PyTorch, Python 3.8	100 vehicles	-
Fan et al. [44]	Simulation	Ubuntu 16.04, 62 GB RAM, 48 CPU cores, 2x NVIDIA GTX 1080 Ti GPUs	PyTorch	100 workers (50 per round)	Bad workers
Huang et al. [47]	Simulation	Laptop Intel Xeon CPU, 32 GB RAM	Custom FL	-	-
Wazzeh et al. [9]	Simulation	16 CPUs @ 2.90 GHz, 16 GB RAM, GTX 1660 Super GPU	FedML GitHub	Randomly selected	-
Wazzeh et al. [10]	Simulation	Machine with 8-core CPU @ 3.59 GHz, 32 GB RAM, Docker containers (2 GB/container), varied CPU capacities (1.5, 2, 2.5)	PyTorch	Randomly selected per cluster	-
Sakka et al. [101]	Simulation	Lenovo ThinkSystem SR950 8S server; VM: Ubuntu 22.04.3 LTS, 16 GB RAM; Docker Engine v26.0.0; containers capped at 2 GB RAM and 1.5 CPU cores	Python 3.10 Custom FL	5 clients	-
Yazdinejad et al. [40]	Simulation	-	PySyft, PyTorch	30, 40, 50, 60	-
Abdur Rahman et al. [56]	Hybrid	NVIDIA Jetson Nano, Raspberry Pi 4, Intel NCS2, Google CORAL TPU, NVIDIA RTX 2080 GPU	PySyft	7 clients	Poisoning attacks, membership inference attacks
Liu et al. [41]	Simulation	Raspberry Pi 3B, Sugon W5801-G10 (CentOS 7.8)	PyTorch 2.4.0, Python 3.7	20, 40, 60, 80, 100	Malicious nodes
Xiong et al. [63]		Intel Xeon(R) CPU E5-2650 v4 @ 2.20 GHz, Linux Ubuntu 20.04, Tesla P40 GPU, 32 G RAM, 2 × 2T HDD	Pytorch	20, 40, 60, 80, 100	Malicious nodes
Ahmed and Anisi [74]	Hybrid	Raspberry Pi 4, IIoT Gateway, Intel Core i5, High-Performance Workstation, Ubuntu 22.04	Custom FL	50 clients	Impersonation, replay, ML-based inference attacks, poisoning
Chen et al. [13]	Simulation	OnePlus-AcePro Smartphone (Android, 8 GB), Huawei Laptop (Ryzen i7-5700U, Windows 10, 8 GB) and Lenovo Desktop (i5-10500, Windows 10, 8 GB)	Custom FL	-	-
Ji et al. [45]	Simulation	Windows 10, Intel i5-10600kf CPU, RTX2080 GPU, 64 GB RAM	TensorFlow	10, 30, 50, 60, 100	Poisoning attacks
Deebak & Hwang [39]	Simulation	Core i5-1035G1 CPU, 16 GB RAM, 2.3 GHz	TensorFlow Federated	3 clients	Poisoning attacks
Feng et al. [42]	Simulation	-	Custom FL	50 clients	Collusion attacks

## Data Availability

No new data were created or analyzed in this study.
